# The CHI3L1-neutrophil axis drives immune suppression and breast cancer metastatic dissemination

**DOI:** 10.1172/jci.insight.199307

**Published:** 2026-02-03

**Authors:** Tarek Taifour, Adéline Massé, Yu Gu, Virginie Sanguin-Gendreau, Dongmei Zuo, Bin Xiao, Emilie Solymoss, Yunyun Shen, Hailey Proud, Sherif Samer Attalla, Vasilios Papavasiliou, Nancy U. Lin, Melissa E. Hughes, Kalie Smith, Chun Geun Lee, Suchitra Kamle, Josie Ursini-Siegel, Jack A. Elias, Peter M. Siegel, Rinath Jeselsohn, William J. Muller

**Affiliations:** 1 McGill University, Division of Experimental Medicine, Department of Medicine, Faculty of Medicine, Montreal, Quebec, Canada.; 2 Rosalind and Morris Goodman Cancer Institute, Montreal, Quebec, Canada.; 3 McGill University, Department of Biochemistry, Faculty of Medicine, Montreal, Quebec, Canada.; 4 Department of Medical Oncology, Dana-Farber Cancer Institute, Boston, Massachusetts, USA.; 5 Brown University, Molecular Biology and Immunology, Providence, Rhode Island, USA.; 6 Hanyang University, Intercollege, Seoul, South Korea.; 7 Lady Davis Institute for Medical Research, Jewish General Hospital, Montreal, Quebec, Canada.

**Keywords:** Immunology, Oncology, Breast cancer, Extracellular matrix, Neutrophils

## Abstract

Immunosuppression and metastasis are critical hallmarks of breast cancer, often linked to poor patient outcomes. The secreted cytokine chitinase-3–like 1 (CHI3L1) is frequently overexpressed in breast cancer samples and promotes an immunosuppressed tumor microenvironment. Notably, CHI3L1 expression is elevated in metastatic patient samples when compared with the matched primary breast tumor. To investigate its role in breast cancer metastasis, we generated an inducible genetically engineered mouse model that overexpresses CHI3L1 in the mammary epithelium. Ectopic expression of CHI3L1 in the polyomavirus middle T (PyMT) mouse model of breast cancer suppressed antitumor immune responses, accelerated mammary tumor onset, and enhanced lung metastasis. Mechanistically, elevated CHI3L1 expression in the mammary epithelium enhanced neutrophil recruitment, which subsequently degraded the extracellular matrix and increased the number of circulating tumor cells. These findings reveal a key mechanism driving metastatic dissemination and argue that therapeutically targeting Chi3l1 could enhance antitumor immunity and suppress metastasis.

## Abstract 2

Metastasis is the leading cause of breast cancer mortality. We demonstrate that STAT3-induced Chi3l1 recruits neutrophils that break extracellular matrix barriers, enabling cancer spread.

## Graphical abstract

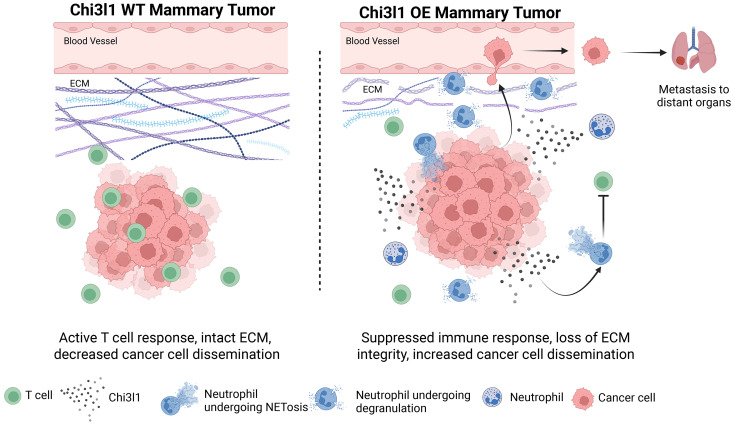

## Introduction

Metastasis is the major cause of morbidity and mortality in breast cancer (1, 2). This multistep process comprises sequential events that include escape from the primary tumor, local invasion of cancer cells, breakdown of the extracellular matrix (ECM), intravasation and survival within the blood vessels, extravasation, and metastatic seeding (3). Throughout this process, the tumor immune microenvironment (TIME) plays a crucial role. Patients with metastatic cancer are often characterized by an immunosuppressive TIME that facilitates metastatic dissemination and hinders the efficacy of immunotherapies such as PD-1/PD-L1 checkpoint blockers (4–6). Thus, elucidating the underlying mechanisms of immunosuppression and metastasis is crucial to identify therapeutic targets and improve patient outcomes (7).

Signal transducer and activator of transcription 3 (STAT3) is an oncogenic transcription factor that suppresses immune responses and promotes metastasis (8, 9). Mammary epithelial ablation of *Stat3* (STAT3 deficient, *Stat3^–/–^*) in a genetically engineered mouse model (GEMM) coexpressing polyomavirus middle T (PyMT) and Cre recombinase (herein referred to as MIC) (10, 11) delays mammary tumor onset and inhibits lung metastases (12). This delay in tumor onset is driven by an active antitumor immune response mediated by CD8^+^ cytotoxic T cells (12).

A critical transcriptional target of STAT3 is the secreted cytokine chitinase-3–like 1 (CHI3L1; YKL-40 in humans) (13–15). CHI3L1 is an immunosuppressive cytokine, a biomarker of breast cancer aggressiveness and tumor grade (13, 14, 16), and is linked to metastasis (17–20). We have previously demonstrated that CHI3L1 induces the formation of neutrophil extracellular traps (NETs), which prevent T cells from infiltrating the tumor epithelium (21). However, whether CHI3L1 is the primary STAT3-driven cytokine responsible for immunosuppression, and its specific role in promoting metastasis, remain to be determined.

In this study, we developed a doxycycline-inducible GEMM that ectopically expresses CHI3L1 in the mammary epithelium (CHI3L1 overexpression, herein referred to as CHI3L1 OE) and crossed it with MIC STAT3-proficient and mammary STAT3-deficient mice. Elevated CHI3L1 expression suppressed the TIME, accelerated mammary tumor onset, and promoted lung metastases in both STAT3-proficient and STAT3-deficient backgrounds, effectively rescuing the tumorigenic defects observed in STAT3-deficient mammary glands. Mechanistically, CHI3L1 recruited neutrophils, which suppressed antitumor immune responses, degraded the ECM, and facilitated cancer cell dissemination. These findings underscore the clinical potential of targeting CHI3L1 to enhance antitumor immune responses and prevent metastatic dissemination.

## Results

### Ectopic expression of CHI3L1 accelerates mammary tumor onset.

To begin dissecting the role of CHI3L1 in breast cancer progression, we confirmed that *Chi3l1* is a direct STAT3-transcriptional target. ChIP in STAT3-proficient and -deficient PyMT^+^ cells demonstrated STAT3 binding to the *Chi3l1* promoter (Supplemental Figure 1, A–D; supplemental material available online with this article; https://doi.org/10.1172/jci.insight.199307DS1). *Chi3l1* promoter fragments were enriched upon STAT3 immunoprecipitation in STAT3-proficient cells but not in STAT3-deficient cells, confirming that *Chi3l1* is a direct STAT3 target gene (Supplemental Figure 1, C and D).

**Figure 1:**
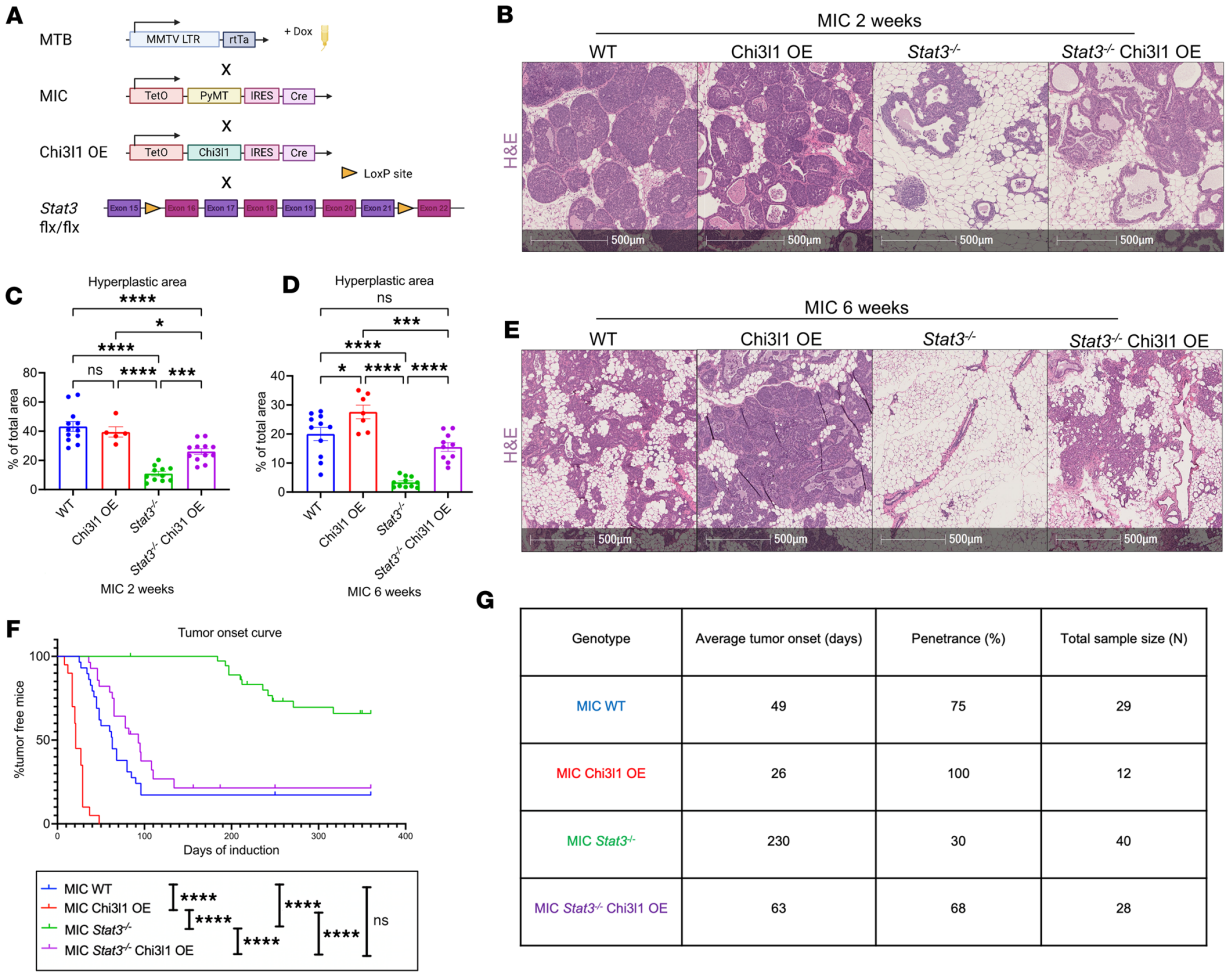
Chi3l1 OE rescues mammary tumor onset in Stat3-deficient MIC mice. (**A**) Schematic representation of the MTB MIC *Stat3^–/–^* Chi3l1 OE GEMM used in this study. Created in BioRender (Muller W, 2026, https://BioRender.com/avc83im). (**B**) Representative hematoxylin and eosin (H&E) staining of WT, Chi3l1 OE, *Stat3^–/–^*, and *Stat3^–/–^* Chi3l1 OE MIC mammary glands at 2 weeks after induction. (**C**) Quantification of hyperplastic area in WT (*n* = 12), Chi3l1 OE (*n* = 5), *Stat3^–/–^* (*n* = 11), and *Stat3^–/–^* Chi3l1 OE (*n* = 12) MIC mammary glands at 2 weeks after induction. Represented as percentage of total mammary gland area. (**D**) Quantification of hyperplastic area in WT (*n* = 11), Chi3l1 OE (*n* = 7), *Stat3^–/–^* (*n* = 11), and *Stat3^–/–^* Chi3l1 OE (*n* = 11) MIC mammary glands at 6 weeks after induction. Represented as percentage of total mammary gland area. (**E**) Representative H&E staining of WT, Chi3l1 OE, *Stat3^–/–^*, and *Stat3^–/–^* Chi3l1 OE MIC mammary glands at 6 weeks after induction. (**F**) Mammary tumor onset in WT (*n* = 29), Chi3l1 OE (*n* = 20), *Stat3^–/–^* (*n* = 40), and *Stat3^–/–^* Chi3l1 OE (*n* = 28) MIC mice, tracked by weekly physical palpation. Analysis by log-rank test. (**G**) Summary of mammary tumor onset and penetrance in WT, Chi3l1 OE, *Stat3^–/–^*, and *Stat3^–/–^* Chi3l1 OE MIC mice. *P < 0.05, ***P < 0.001, ****P < 0.0001 by 1-way ANOVA with Tukey’s post hoc test. Scale bars: 500 μm.

We then generated an inducible GEMM that ectopically overexpresses CHI3L1 in the mammary epithelial cells (CHI3L1 OE). This strain was crossed to our STAT3-deficient MIC GEMM to assess whether ectopic CHI3L1 expression can rescue defects in cancer progression observed upon *Stat3* ablation (Figure 1A). We validated the levels of CHI3L1 in the mammary glands through immunoblot analysis (Supplemental Figure 2, A–D). Consistent with our previous work (21), STAT3-deficient mammary glands exhibited significantly reduced CHI3L1 levels. However, ectopic CHI3L1 expression restored CHI3L1 levels in STAT3-deficient glands to those observed in STAT3-proficient glands (Supplemental Figure 2, A–D). Immunofluorescent staining and RNA fluorescence in situ hybridization (RNA FISH) further verified that *Chi3l1* expression and ablation of STAT3 were specific to the epithelial cells, with no impact on stromal levels of *Chi3l1* or STAT3 (Supplemental Figure 2, E–J).

Furthermore, we examined several well-established STAT3 target genes (22) by qPCR to verify their downregulation in STAT3-deficient mammary glands. These targets included *Cxcl1* (C-X-C motif chemokine ligand 1), *Cxcl5* (C-X-C motif chemokine ligand 5), *Gata2* (GATA-binding protein 2), *Vegf* (vascular endothelial growth factor), *Tnfrsf1a* (tumor necrosis factor receptor superfamily member 1A), *Cebpd* (CAAT/enhancer-binding protein δ), *Saa2* (serum amyloid A2), *Bcl3* (B cell lymphoma 3 protein), *Il1B* (interleukin-1β), *MMP2* (matrix metalloproteinase 2), *Gcsf* (granulocyte colony-stimulating factor), and *Gmcf* (granulocyte-macrophage colony-stimulating factor). As expected, they were significantly reduced upon *Stat3* ablation but remained unchanged in CHI3L1 OE mammary glands (Supplemental Figure 3, A–N). These findings confirm that any phenotypes observed are attributable specifically to CHI3L1 overexpression, rather than upregulation of other STAT3 targets.

Histological analyses of mammary glands 2 and 6 weeks after induction revealed a reduction in ductal intraepithelial neoplasia upon loss of *Stat3* (Figure 1, B–E). However, re-expression of CHI3L1 rescued this defect and increased ductal intraepithelial neoplasia in STAT3-deficient glands (Figure 1, B–E). Interestingly, ectopic expression of CHI3L1 in STAT3-proficient glands also increased hyperplasia at 6 weeks after induction, indicating accelerated tumor progression (Figure 1, D and E). Consistently, we observed an earlier onset of palpable mammary tumors in CHI3L1 OE STAT3-proficient mice compared with wild-type (WT) STAT3-proficient mice (Figure 1, F and G). While STAT3-deficient mice had a drastic delay in tumor onset and decreased tumor penetrance, CHI3L1 OE *Stat3^–/–^* mice developed tumors at a similar time and penetrance compared with STAT3-proficient mice (Figure 1, F and G). Thus, elevated expression of CHI3L1 rescues the tumorigenic defects observed upon loss of *Stat3*, indicating that CHI3L1 is critical for mammary tumor initiation and progression. It also demonstrates that CHI3L1 is the major STAT3-driven cytokine promoting cancer progression in this model.

### CHI3L1 creates an immune-suppressed TIME.

**Figure 2:**
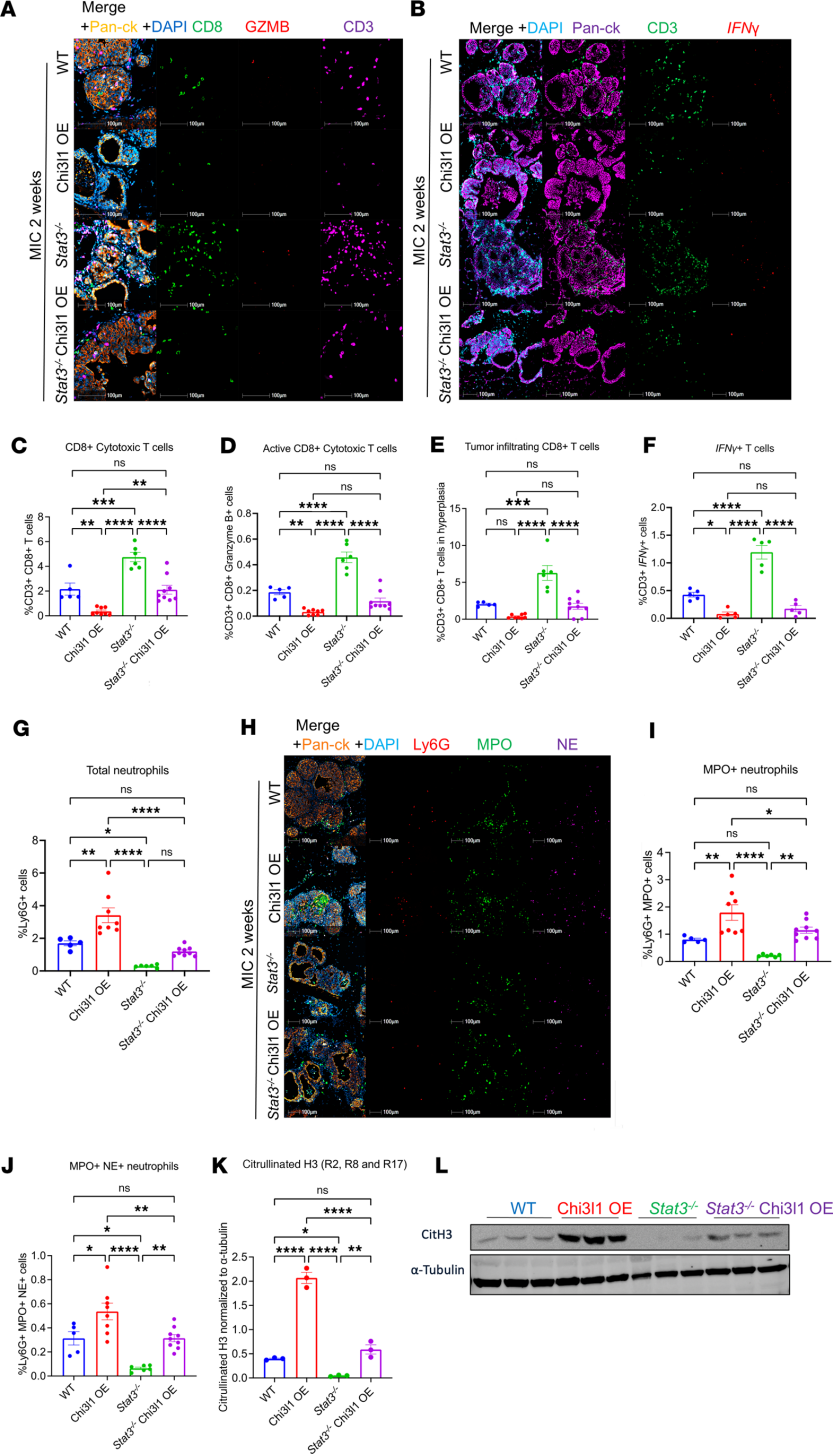
Chi3l1 OE suppresses antitumor immune responses. (**A**) Staining of WT, Chi3l1 OE, *Stat3^–/–^*, and *Stat3^–/–^* Chi3l1 OE MIC mammary glands at 2 weeks after induction for CD8, granzyme B (GZMB), CD3, Pan-CK, and DAPI. (**B**) RNA FISH against IFN-γ with staining for CD3, Pan-CK, and DAPI on mammary tissue from WT, Chi3l1 OE, *Stat3^–/–^*, and *Stat3^–/–^* Chi3l1 OE MIC mammary glands at 2 weeks after induction. (**C**–**G**) Quantification of total CD3^+^CD8^+^, CD3^+^CD8^+^GZMB^+^, and tumor-infiltrating CD3^+^CD8^+^ T cells, CD3^+^IFN-γ^+^ cells, and Ly6G^+^ cells in WT (*n* = 5), Chi3l1 OE (*n* = 8), *Stat3^–/–^* (*n* = 6), and *Stat3^–/–^* Chi3l1 OE (*n* = 9) MIC mammary glands at 2 weeks after induction. (**H**) Staining of WT, Chi3l1 OE, *Stat3^–/–^*, and *Stat3^–/–^* Chi3l1 OE MIC mammary glands at 2 weeks after induction using antibodies against Ly6G, MPO, NE, pan-CK, and DAPI. (**I** and **J**) Quantification of total Ly6G^+^MPO^+^ cells and Ly6G^+^MPO^+^NE^+^ cells in WT (*n* = 5), Chi3l1 OE (*n* = 8), *Stat3^–/–^* (*n* = 6), and *Stat3^–/–^* Chi3l1 OE (*n* = 9) MIC mammary glands at 2 weeks after induction. (**K**) Quantification of CitH3 immunoblots normalized to α-tubulin. (**L**) Immunoblots for CitH3 and α-tubulin on WT (*n* = 3), Chi3l1 OE (*n* = 3), *Stat3^–/–^* (*n* = 3), and *Stat3^–/–^* Chi3l1 OE (*n* = 3) MIC mammary glands at 2 weeks after induction. *P < 0.05, **P < 0.01, ***P < 0.001, ****P < 0.0001 by 1-way ANOVA with Tukey’s post hoc test. Scale bars: 100 μm.

*Stat3* ablation in the MIC GEMM results in active antitumor immune responses that clear hyperplastic lesions (12). To investigate whether CHI3L1 rescued mammary tumorigenesis by suppressing these immune responses, we performed multiplex immunofluorescent staining for T cell markers in mammary glands at 2 weeks after induction. Elevated CHI3L1 expression in STAT3-proficient glands resulted in a reduction in cytotoxic CD8^+^ T cell infiltration and impaired activation, as assessed by reduced granzyme B (GZMB) and interferon-γ (*IFNG*) expression (Figure 2, A–F). Similarly, CHI3L1 expression led to decreased recruitment and activation of CD4^+^ T helper cells (Supplemental Figure 4, A–D). T cell infiltration into the tumor nest was also inhibited in CHI3L1 OE mammary glands (Figure 2E and Supplemental Figure 4D). As previously demonstrated, *Stat3* ablation resulted in increased recruitment and activation of CD8^+^ and CD4^+^ T cells (Figure 2, A–F, and Supplemental Figure 4, A–D). However, CHI3L1 expression in STAT3-deficient mammary glands reversed this phenotype, resulting in a degree of T cell infiltration similar to that in STAT3-proficient glands (Figure 2, A–F, and Supplemental Figure 4, A–D).

We have previously shown that CHI3L1 promotes neutrophil recruitment and NETosis, which blocks T cell infiltration (21). We thus interrogated neutrophil levels in the mammary glands through staining and immunoblot analyses. LY6G (lymphocyte antigen 6 complex locus G6D), myeloperoxidase (MPO), and neutrophil elastase (NE) were used as neutrophil markers, while citrullinated H3 (CitH3) was used as a marker of NETosis. Ectopic expression of CHI3L1 in STAT3-proficient glands caused a significant increase in overall neutrophil infiltration as well as enhanced NETosis (Figure 2, G–L). While STAT3-deficient glands exhibited reduced neutrophil recruitment, these changes were abrogated upon CHI3L1 re-expression, restoring neutrophils and NETosis to baseline levels observed in STAT3-proficient glands (Figure 2, G–L).

Previous studies have demonstrated that CHI3L1 promotes macrophage recruitment and polarization into the pro-tumorigenic M2 state (18, 23, 24). To assess macrophages in our models, we performed immunofluorescent staining using F4/80 as a murine macrophage marker, CD206 as a marker of M2 macrophages, and phosphorylated STAT1 (p-STAT1) as a marker of antitumor M1 macrophages. The results revealed no significant differences in macrophage levels or their polarization between WT and CHI3L1 OE glands (Supplemental Figure 4, E–I). Consistent with our previous study (12), *Stat3^–/–^* tumors were enriched with total macrophages and M1 macrophages (Supplemental Figure 4, E–I). These phenotypes remained consistent and were not reversed in *Stat3^–/–^* CHI3L1 OE glands (Supplemental Figure 4, E–I). Overall, these data indicate that the phenotypes observed in CHI3L1 OE mice are not driven by macrophages.

All changes to T cell, neutrophil, and macrophage populations were confirmed using fluorescence-activated cell sorting (FACS) analysis at 2 weeks after induction (Supplemental Figure 5, A–O). Our FACS panel also revealed that CHI3L1 overexpression suppressed natural killer (NK) cell levels in the mammary glands (Supplemental Figure 5H). While STAT3-deficient lesions were enriched with NK cells, their levels decreased significantly in STAT3-deficient CHI3L1 OE glands (Supplemental Figure 5H). Interestingly, we observed no difference in monocyte populations in any group (Supplemental Figure 5, M and N), indicating that they were not contributing to our phenotypes.

Because the effects of CHI3L1 OE were more pronounced at 6 weeks after induction compared with 2 weeks (Figure 1, D and E), we also performed immunofluorescent staining for various immune populations at 6 weeks after induction. Consistent with our data at the 2-week time point, CHI3L1 OE mammary glands were enriched with neutrophils and exhibited significantly reduced infiltration of antitumor CD8^+^ cytotoxic T cells and CD4^+^ T helper cells compared with STAT3-proficient mammary glands (Supplemental Figure 6, A–I). STAT3-deficient glands showed the opposite pattern, with significantly fewer neutrophils and increased T cell infiltration (Supplemental Figure 6, A–I). Importantly, CHI3L1 OE glands reversed these effects, restoring neutrophil abundance to levels comparable to those of WT glands and suppressing T cell infiltration (Supplemental Figure 6, A–I). We also observed no significant differences in macrophage populations across the groups (Supplemental Figure 6, J–L). Thus, these data argue that CHI3L1 is a major immunosuppressive cytokine that supports tumor progression by elevating neutrophil abundance while suppressing T cell recruitment and activation.

### Ectopic expression of CHI3L1 drives cancer progression through neutrophils.

**Figure 3:**
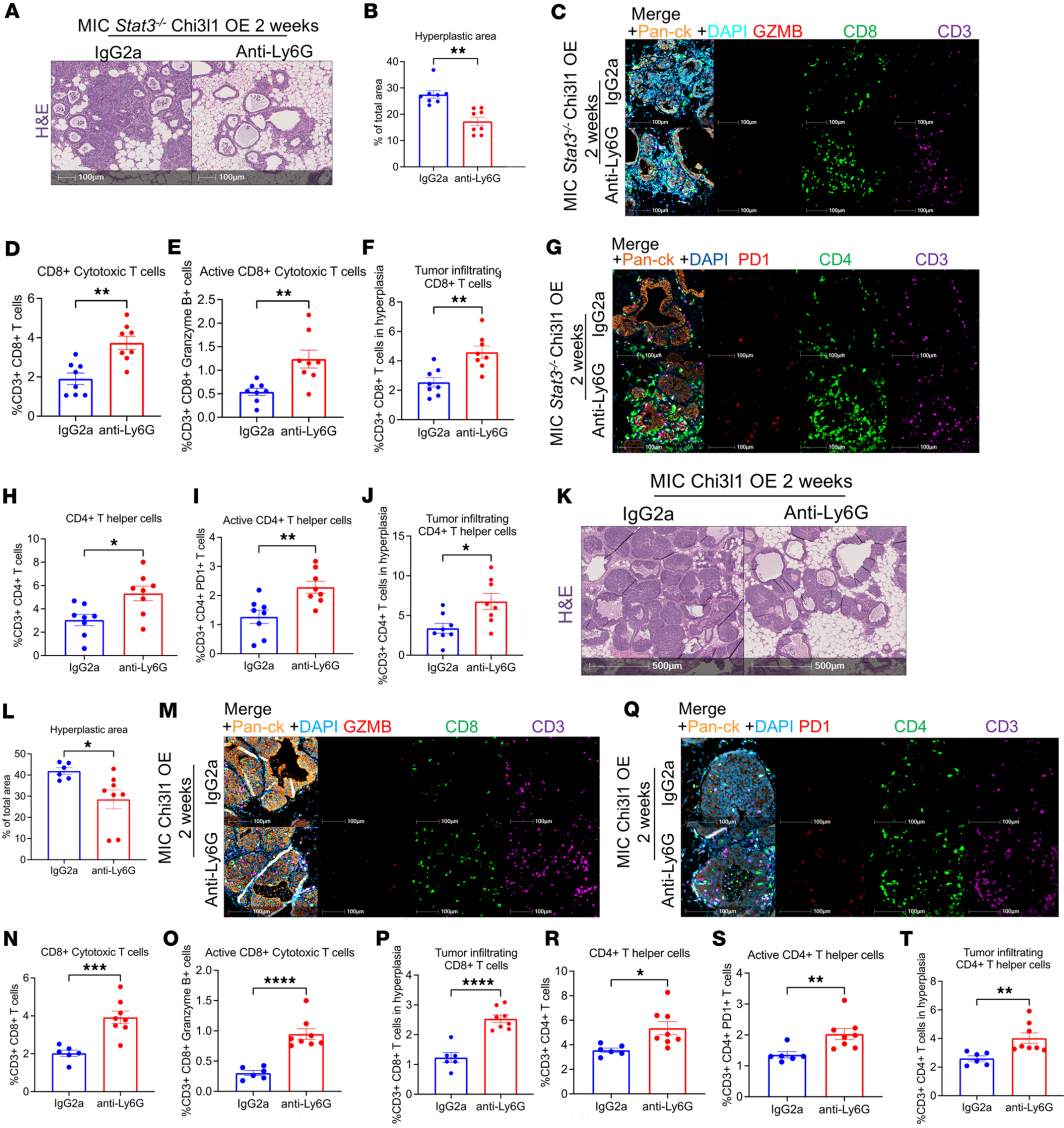
Neutrophil depletion abrogates the effects of Chi3l1 OE. (**A**) H&E staining of mammary glands from IgG2a- and anti-Ly6G–treated MIC *Stat3^–/–^* Chi3l1 OE mice at 2 weeks after induction. (**B**) Quantification of hyperplastic area in IgG2a-treated (*n* = 8) and anti-Ly6G–treated (*n* = 8) MIC *Stat3^–/–^* Chi3l1 OE mice. Represented as percentage of mammary gland area. (**C**) Staining of mammary tissue from MIC *Stat3^–/–^* Chi3l1 OE mice treated with anti-Ly6G or IgG2a for 2 weeks, for GZMB, CD8, CD3, pan-CK, and DAPI. (**D**–**F**) Quantification of CD3^+^CD8^+^, CD3^+^CD8^+^GZMB^+^, and tumor-infiltrating CD3^+^CD8^+^ T cells in IgG2a-treated (*n* = 8) and anti-Ly6G–treated (*n* = 8) MIC *Stat3^–/–^* Chi3l1 OE mammary glands. (**G**) Staining of mammary tissue from MIC *Stat3^–/–^* Chi3l1 OE mice treated with anti-Ly6G or IgG2a, for PD-1, CD4, CD3, pan-CK, and DAPI. (**H**–**J**) Quantification of CD3^+^CD4^+^, CD3^+^CD4^+^PD-1^+^, and tumor-infiltrating CD3^+^CD4^+^ T cells in IgG2a-treated (*n* = 8) and anti-Ly6G–treated (*n* = 8) MIC *Stat3^–/–^* Chi3l1 OE mammary glands. (**K**) H&E staining of mammary glands from IgG2a- and anti-Ly6G–treated MIC Chi3l1 OE mice at 2 weeks after induction. (**L**) Quantification of hyperplastic area in IgG2a-treated (*n* = 6) and anti-Ly6G–treated (*n* = 8) MIC Chi3l1 OE mice. Represented as percentage of mammary gland area. (**M**) Staining of mammary tissue from MIC Chi3l1 OE mice treated with anti-Ly6G or IgG2a for 2 weeks, for GZMB, CD8, CD3, pan-CK, and DAPI. (**N**–**P**) Quantification of CD3^+^CD8^+^, CD3^+^CD8^+^ GZMB^+^, and tumor-infiltrating CD3^+^CD8^+^ T cells in IgG2a-treated (*n* = 6) and anti-Ly6G–treated (*n* = 8) MIC Chi3l1 OE mammary glands. (**Q**) Staining of mammary tissue from MIC Chi3l1 OE mice treated with anti-Ly6G or IgG2a for 2 weeks, for PD-1, CD4, CD3, pan-CK, and DAPI. (**R**–**T**) Quantification of CD3^+^CD4^+^, CD3^+^CD4^+^PD1^+^, and tumor-infiltrating CD3^+^CD4^+^ T cells in IgG2a-treated (*n* = 6) and anti-Ly6G–treated (*n* = 8) MIC Chi3l1 OE mammary glands. *P < 0.05, **P < 0.01, ***P < 0.001, ****P < 0.0001 by unpaired Student’s *t* test. Scale bars: 100 μm in **A**, **C**, **G**, **M**, and **Q**; 500 μm in **K**.

To explore whether the ability of CHI3L1 to rescue the STAT3-deficient phenotypes required neutrophil functions, we depleted neutrophils in STAT3-deficient CHI3L1 OE glands through anti-LY6G treatment (Supplemental Figure 7A). Successful depletion was confirmed through FACS for circulating neutrophils as well as immunofluorescence and immunoblotting of mammary gland tissues (Supplemental Figure 7, B–J). Histological analyses of the mammary glands at 2 weeks after induction revealed that neutrophil depletion markedly reduced ductal intraepithelial neoplasia in comparison with IgG2a-treated control mice, effectively nullifying the effects of CHI3L1 (Figure 3, A and B). Immunofluorescent staining further demonstrated that anti-LY6G–treated glands were enriched with activated CD8^+^ T cells and CD4^+^ T helper cells compared with IgG2a-treated glands (Figure 3, C–J). Tumor infiltration of these T cell subsets was also increased after neutrophil depletion (Figure 3, F and J). Therefore, neutrophil depletion reverts the glands to a state similar to that observed in STAT3-deficient glands.

Similar results were obtained in CHI3L1 OE Stat3-proficient glands, where neutrophil depletion (Supplemental Figure 7, K–S) caused a significant reduction in ductal intraepithelial neoplasia (Figure 3, K and L). Furthermore, the total number and activation of cytotoxic T cells as well as T helper cells were increased after neutrophil depletion (Figure 3, M–T). Thus, CHI3L1 exerts its tumorigenic effects by recruiting neutrophils that suppress T cell infiltration and activation, leading to accelerated mammary tumor onset.

### CHI3L1-mediated neutrophil recruitment drives metastatic dissemination.

**Figure 4:**
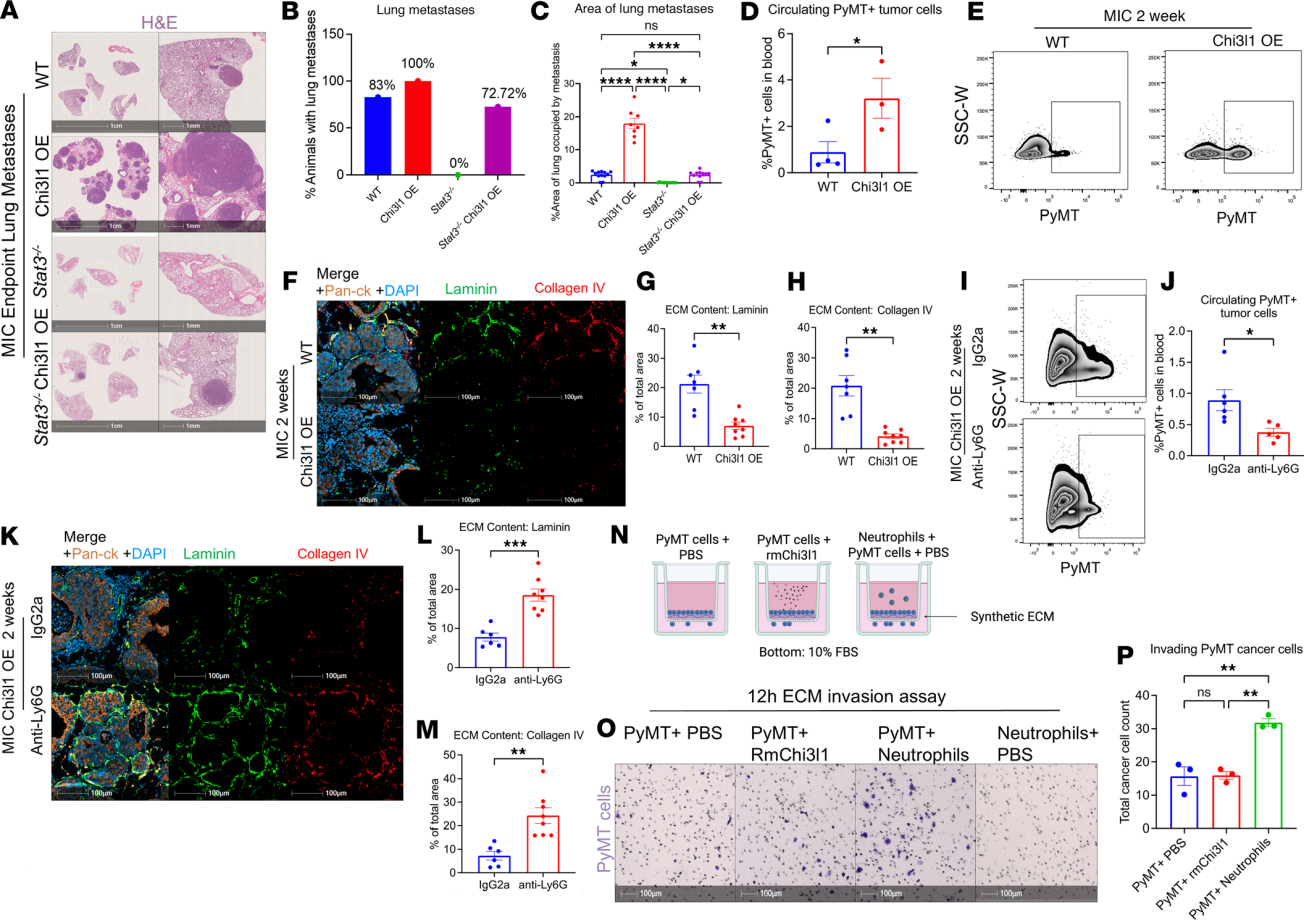
Chi3l1 OE enhances metastatic dissemination through neutrophil-mediated ECM remodeling. (**A**) H&E staining of WT, Chi3l1 OE, *Stat3^–/–^*, and *Stat3^–/–^* Chi3l1 OE MIC lungs at mammary tumor endpoint. (**B**) Percentage of WT (*n* = 12), Chi3l1 OE (*n* = 8), *Stat3^–/–^* (*n* = 13), and *Stat3^–/–^* Chi3l1 OE (*n* = 11) MIC mice with pulmonary metastases. (**C**) Quantification of metastatic area (percent total lung area) in WT (*n* = 12), Chi3l1 OE (*n* = 8), *Stat3^–/–^* (*n* = 13), and *Stat3^–/–^* Chi3l1 OE (*n* = 11) MIC lungs. (**D**) Quantification of PyMT^+^ cells (percent live cells in blood) of WT (*n* = 4) and Chi3l1 OE (*n* = 3) MIC mice. (**E**) FACS for circulating PyMT^+^ cells in WT and Chi3l1 OE MIC blood at 2 weeks after induction. (**F**) Staining of WT and Chi3l1 OE MIC mammary glands at 2 weeks after induction for laminin, collagen IV, pan-CK, and DAPI. (**G** and **H**) Quantification of area occupied by laminin or collagen IV (percent total mammary gland area) in WT (*n* = 7) and Chi3l1 OE (*n* = 8) MIC mammary glands. (**I**) FACS for circulating PyMT^+^ cells in blood of Chi3l1 OE MIC mice after anti-Ly6G or IgG2a treatment for 2 weeks. (**J**) Quantification of PyMT^+^ cells (percent live cells in the blood) of Chi3l1 OE MIC mice treated with IgG2a (*n* = 6) or anti-Ly6G (*n* = 5). (**K**) Staining of mammary tissue from MIC Chi3l1 OE mice treated with anti-Ly6G or IgG2a for laminin, collagen IV, pan-CK, and DAPI. (**L** and **M**) Quantification of area occupied by laminin and collagen IV (percent mammary gland area) in IgG2a-treated (*n* = 6) and anti-Ly6G–treated (*n* = 8) MIC Chi3l1 OE mammary glands. (**N**) Schematic of Transwell invasion assay through synthetic ECM. Created in BioRender (Muller W, 2026, https://BioRender.com/4lhz2a3). (**O**) ECM-invading PyMT^+^ cells with PBS, rmChi3l1, or murine neutrophils, stained with crystal violet. Neutrophils + PBS (no PyMT cells) is shown as negative control. (**P**) Quantification of invading PyMT^+^ cells. *n* = 3 technical replicates for each condition. *P < 0.05, **P < 0.01, ***P < 0.001, ****P < 0.0001 by 1-way ANOVA with Tukey’s post hoc test (**C** and **P**) or by unpaired Student’s *t* test (for **D**, **G**, **H**, **J**, **L**, and **M**). Scale bars: 1 cm in **A**, left panels; 1 mm in **A**, right panels; 100 μm in **F**, **K**, and **O**.

Another critical observation from STAT3-deficient mice is that they are devoid of lung metastases (12). Since CHI3L1 expression suppresses the immune system and rescues tumor formation, we next investigated its impact on lung metastasis. Lungs were collected from mammary tumor-bearing mice at the same endpoint tumor burden and were analyzed for metastatic lesions. Ectopic expression of CHI3L1 increased both the incidence and extent of lung metastases in STAT3-proficient and STAT3-deficient backgrounds (Figure 4, A–C). Notably, while none of the STAT3-deficient mice exhibited lung metastases, 72% of the STAT3-deficient CHI3L1 OE mice developed metastatic lesions (Figure 4, A–C), indicating that CHI3L1 overexpression completely rescues metastatic progression in STAT3-deficient mice.

We next assessed whether CHI3L1 overexpression caused earlier dissemination of cancer cells. FACS analysis demonstrated an increase in PyMT^+^ circulating tumor cells (CTCs) at 2 weeks after induction in CHI3L1 OE mice compared with WT mice (Figure 4, D and E). Furthermore, the increase in CTCs led to an earlier onset of lung metastases, with 60% of the mice developing metastatic lesions at 6 weeks after induction, a time point at which none of the WT mice exhibited metastases (Supplemental Figure 8, A and B). Since early dissemination of cancer cells correlates with ECM loss (25), we next examined ECM levels in CHI3L1 OE mammary glands. Immunofluorescent staining for the ECM markers laminin and collagen IV revealed that elevated CHI3L1 expression in STAT3-proficient glands significantly reduced ECM levels, assessed by area occupied by laminin or collagen IV at 2 weeks and 6 weeks (Figure 4, F–H, and Supplemental Figure 8, C and D). Thus, expression of CHI3L1 facilitates cancer cell dissemination by remodeling the ECM.

As our data demonstrate that expression of CHI3L1 suppresses the immune system through neutrophils, we next investigated whether neutrophils contribute to CHI3L1-driven cancer cell dissemination. Neutrophil depletion through anti-LY6G treatment significantly reduced PyMT^+^ CTCs in both CHI3L1 OE STAT3-proficient and STAT3-deficient mice (Figure 4, I and J, and Supplemental Figure 8, F and G). Taken together, these results argue that CHI3L1 enhances CTCs through the recruitment of neutrophils.

Previous studies have shown that neutrophils remodel the ECM by secreting proteases, collagenases, and granular components (26, 27). Consistent with this concept, immunofluorescent staining revealed that neutrophil depletion restored ECM integrity in CHI3L1 OE STAT3-proficient glands (Figure 4, K–M). To independently confirm the role of neutrophils in ECM remodeling and cancer cell invasion, we performed an in vitro Transwell invasion assay, culturing PyMT^+^ cancer cells on a synthetic ECM in the presence of PBS or recombinant murine CHI3L1 (rmCHI3L1) or cocultured with murine neutrophils (Figure 4N). After 12 hours, cancer cells stimulated with rmCHI3L1 or PBS failed to invade through the synthetic ECM (Figure 4, O and P). However, coculture with neutrophils significantly increased PyMT^+^ cell invasion (Figure 4, O and P). To evaluate whether neutrophils contribute to ECM degradation, we performed in vitro ECM degradation assays in which PyMT^+^ cancer cells were cultured alone or with murine neutrophils on synthetic ECM. Immunofluorescent staining revealed a marked reduction in laminin coverage when cancer cells were cocultured with neutrophils (Supplemental Figure 8, H and I). In addition, coculture on a fluorescently labeled collagen IV matrix caused a significant increase in fluorescence, which occurs only upon collagen IV degradation, indicating extensive matrix breakdown (Supplemental Figure 8, J and K).

Because neutrophils are known to promote cancer cell proliferation (28), it was critical to rule out increased proliferation as a cause of the increased cancer cell invasion. To test this, we stained PyMT^+^ cancer cells cultured alone or with neutrophils for proliferation markers Ki67 and 5-ethynyl-2′-deoxyuridine (EdU). The results revealed no significant differences in KI67^+^ cells and a decrease in EdU positivity when cancer cells were cocultured with neutrophils (Supplemental Figure 8, L–O). Consistent with these findings, an IncuCyte proliferation assay demonstrated that PyMT cancer cells proliferated at a slower rate when cultured in neutrophil-conditioned medium compared with control medium (Supplemental Figure 8, P and Q). These data indicate that our phenotypes were not driven by increased proliferation. Overall, these findings demonstrate that CHI3L1 promotes metastatic dissemination through neutrophil-mediated ECM degradation.

### Neutrophils remodel the ECM through NETosis and degranulation.

**Figure 5:**
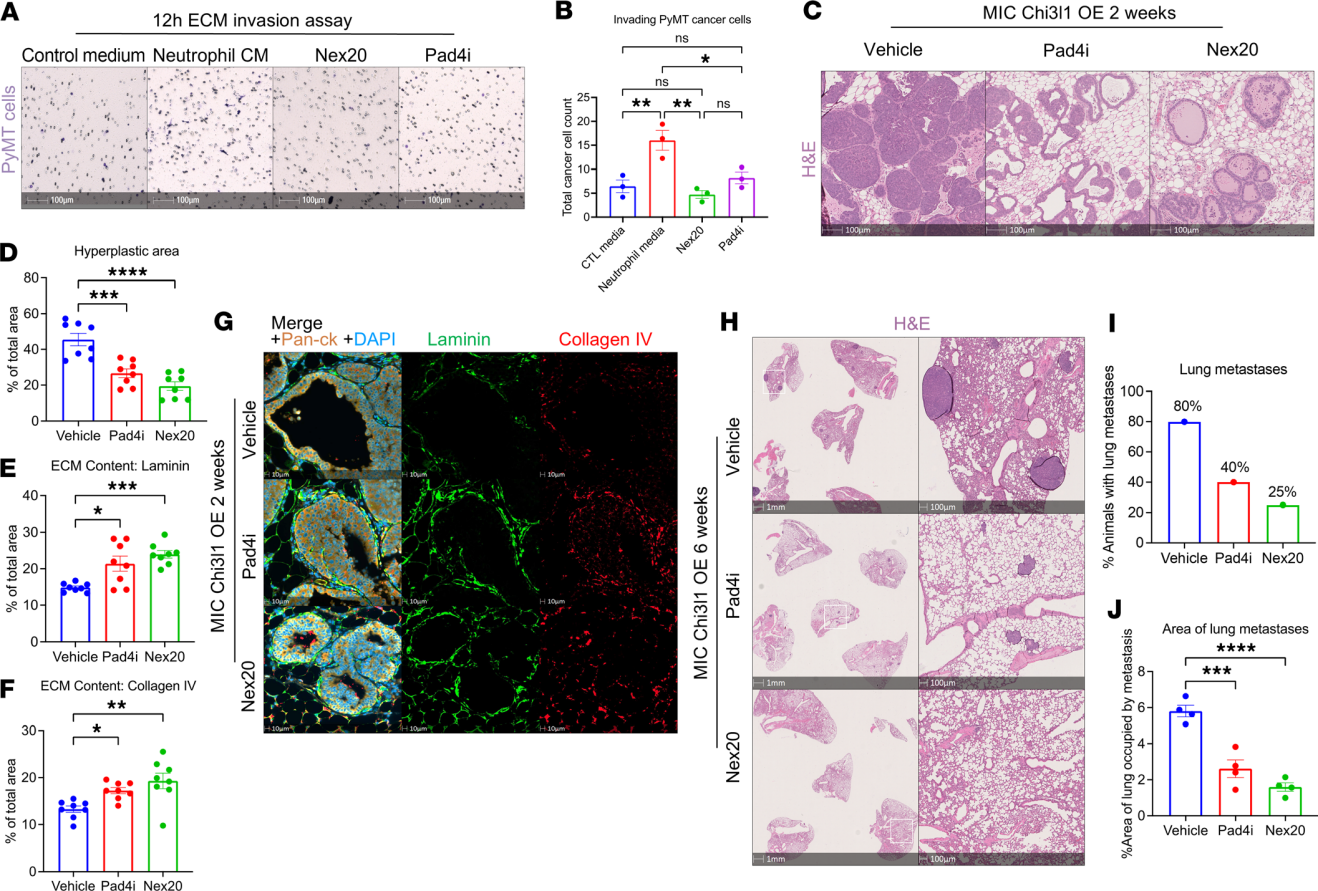
Neutrophil degranulation and NETosis promote ECM remodeling. (**A**) Representative images of ECM-invading PyMT^+^ cells with control medium or neutrophil-conditioned medium (Neutrophil CM) treated with vehicle, Nex20, or Pad4i, stained with crystal violet. (**B**) Quantification of invading PyMT^+^ cells (*n* = 3 technical replicates) in **A**. (**C**) H&E staining of mammary glands from vehicle-, Pad4i-, or Nex20-treated MIC Chi3l1 OE mice at 2 weeks after induction. (**D**) Quantification of hyperplastic area in vehicle-treated (*n* = 8), Pad4i-treated (*n* = 8), and Nex20-treated (*n* = 8) MIC Chi3l1 OE mice at 2 weeks after induction. Represented as percentage of total mammary gland area. (**E** and **F**) Quantification of area occupied by laminin or collagen IV in Pad4i-treated (*n* = 8), Nex20-treated (*n* = 8), or vehicle-treated (*n* = 8) MIC Chi3l1 OE mammary glands at 2 weeks after induction. Represented as percentage of total mammary gland area. (**G**) Staining of mammary tissue from MIC Chi3l1 OE mice treated with Pad4i, Nex20, or vehicle control for 2 weeks, using antibodies against laminin, collagen IV, pan-CK, and DAPI. (**H**) Representative H&E staining of Chi3l1 OE MIC lungs treated with Pad4i, Nex20, or vehicle control for 6 weeks. (**I**) Percentage of Chi3l1 OE MIC mice with pulmonary metastases after treatment with Pad4i, Nex20, or vehicle control for 6 weeks. (**J**) Quantification of total pulmonary metastatic area in Chi3l1 OE MIC lungs after treatment with Pad4i (*n* = 4), Nex20 (*n* = 4), or vehicle control (*n* = 4) for 6 weeks. Represented as percentage of total lung area. *P < 0.05, **P < 0.01, ***P < 0.001, ****P < 0.0001 by 1-way ANOVA with Dunnett’s post hoc test. Scale bars: 100 μm in **A** and **C**; 10 μm in **G**; 1 mm in **H**, left panels; 100 μm in **H**, right panels.

To further define neutrophil functions that drive this phenotype, we performed the in vitro Transwell invasion assay using PyMT^+^ cells cultured in conditioned medium derived from murine neutrophils treated with vehicle, Nexinhib20 (Nex20) to inhibit degranulation (29), or peptidyl arginine deiminase 4 inhibitor (Pad4i; GSK484) to inhibit NETosis (30). While neutrophil-conditioned medium stimulated cancer cell invasion, this effect was significantly reduced in medium from Nex20 or Pad4i-treated neutrophils (Figure 5, A and B). Thus, both neutrophil degranulation and NETosis contribute to ECM remodeling.

To further validate these findings in vivo, we treated CHI3L1 OE STAT3-proficient mice with Nex20, Pad4i, or vehicle for 2 weeks. Immunofluorescent staining confirmed that neither treatment affected LY6G^+^ neutrophil levels (Supplemental Figure 9, A and B). Importantly, each inhibitor specifically blocked its target neutrophil function. Pad4i treatment significantly reduced CitH3 levels, confirming effective inhibition of NETosis, while Nex20 treatment significantly decreased serum MPO and NE levels, consistent with inhibition of neutrophil degranulation (Supplemental Figure 9, C–F).

Histological analyses of the mammary glands at 2 weeks after induction revealed that treatment with either Pad4i or Nex20 significantly reduced ductal intraepithelial neoplasia in comparison with vehicle-treated mice (Figure 5, C and D). Furthermore, immunofluorescent staining demonstrated robust retention of laminin and collagen IV after inhibition of NETosis or neutrophil degranulation (Figure 5, E–G). Together, these findings demonstrate that the ECM defects observed in CHI3L1-expressing mammary glands are driven by neutrophil-mediated ECM degradation through both NETosis and degranulation.

To determine whether NETosis and degranulation contribute to CHI3L1-driven metastatic dissemination, we treated STAT3-proficient CHI3L1 OE mice with either Pad4i or Nex20 for 6 weeks. After confirmation of successful inhibition of NETosis and neutrophil degranulation (Supplemental Figure 9, G–J), lungs were collected to assess metastatic burden. Histological analyses demonstrated that both Pad4i- and Nex20-treated mice exhibited a significant reduction in lung metastasis compared with vehicle-treated controls (Figure 5, H–J). Thus, our findings confirm that the neutrophil-mediated ECM degradation drives accelerated metastatic dissemination in CHI3L1 OE mice.

CHI3L1 OE mammary glands also exhibit a significant reduction in CD8^+^ T cells, key antitumor effectors that kill cancer cells and restrict metastasis (31). To test whether loss of CD8^+^ T cells contributes to enhanced metastasis observed in CHI3L1 OE mice, we depleted CD8^+^ T cells in STAT3-proficient mice through anti-CD8 antibody treatment. Successful depletion was confirmed through FACS for circulating T cells as well as immunofluorescent staining on the mammary glands (Supplemental Figure 10, A–G). Expression of CD3 and CD8 as well as GZMB was all markedly reduced in anti-CD8–treated glands compared with IgG2b-treated controls (Supplemental Figure 10, D–G). Levels of CD4^+^ T helper cells were unchanged (Supplemental Figure 10, H and I). However, FACS analysis for CTCs revealed no significant difference between anti-CD8– and control-treated mice (Supplemental Figure 10, J and K), indicating that loss of CD8^+^ T cells does not contribute to the increased tumor cell dissemination seen in CHI3L1 OE mice. Consistent with these results, immunofluorescent staining revealed that CD8 depletion did not impact ECM levels (Supplemental Figure 10, L–N) or neutrophil levels (Supplemental Figure 10, O–R), which may explain why the levels of CTCs remained unchanged.

Given the role of CHI3L1 in immunosuppression, we next examined the TIME of metastatic lesions in STAT3-proficient CHI3L1 OE lungs compared with STAT3-proficient controls. Immunofluorescent staining for various T cell, neutrophil, and macrophage markers revealed no significant difference in the levels of any of these immune populations (Supplemental Figure 11, A–M). Furthermore, expression levels of *Chi3l1*, *Stat3*, and other immunomodulatory chemokines such as *Il1B*, *Gcsf*, and *Gmcsf* were unchanged in CHI3L1 OE metastatic lesions compared with WT controls (Supplemental Figure 11, N–R). These observations indicate that the enhanced metastatic content seen in CHI3L1 OE mice is driven through neutrophil-mediated ECM degradation that facilitates early metastatic dissemination.

### CHI3L1 is a therapeutic target and biomarker of breast cancer metastasis.

**Figure 6:**
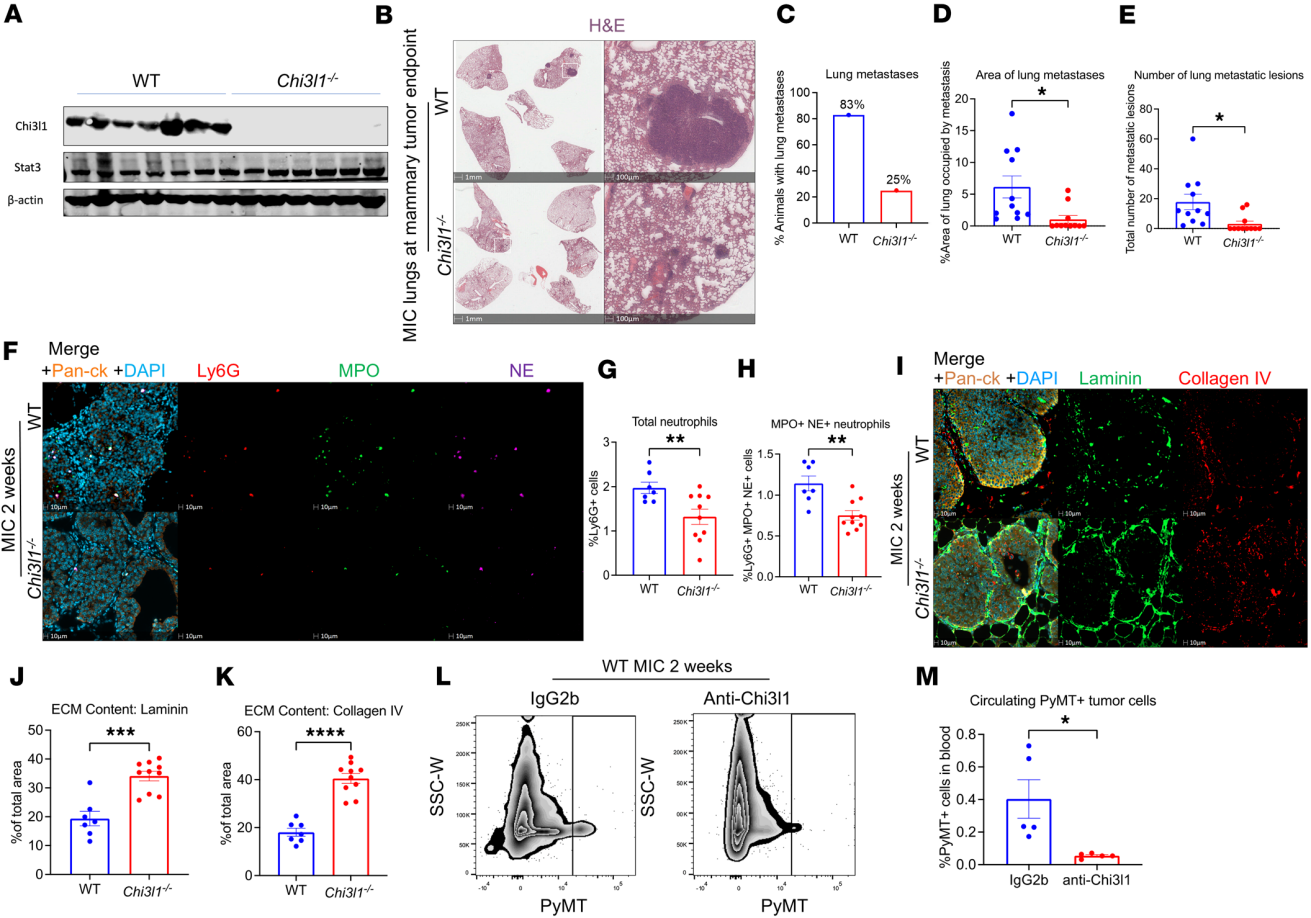
Chi3l1 ablation inhibits breast cancer metastasis. (**A**) Immunoblots for Chi3l1, Stat3, and α-tubulin on WT (*n* = 7) and *Chi3l1^–/–^* (*n* = 6) MIC mammary endpoint tumors. (**B**) Representative H&E staining of WT and *Chi3l1^–/–^* MIC lungs at mammary tumor endpoint. (**C**) Percentage of WT (*n* = 11) and *Chi3l1^–/–^* (*n* = 11) MIC mice with pulmonary metastases. (**D**) Quantification of total pulmonary metastatic area in WT (*n* = 11) and *Chi3l1^–/–^* (*n* = 11) MIC lungs. Represented as percentage of total lung area. (**E**) Number of lung metastatic lesions in WT (*n* = 11) and *Chi3l1^–/–^* (*n* = 11) MIC lungs. (**F**) Staining of WT and *Chi3l1^–/–^* MIC mammary glands at 2 weeks after induction, for Ly6G, MPO, NE, pan-CK, and DAPI. (**G** and **H**) Quantification of Ly6G^+^ cells and Ly6G^+^MPO^+^NE^+^ cells in WT (*n* = 7) and *Chi3l1^–/–^* (*n* = 10) MIC mammary glands at 2 weeks after induction. (**I**) Staining of WT and *Chi3l1^–/–^* MIC mammary glands at 2 weeks after induction, for laminin, collagen IV, pan-CK, and DAPI. (**J** and **K**) Quantification of area occupied by laminin or collagen IV in WT (*n* = 7) and *Chi3l1^–/–^* (*n* = 10) MIC mammary glands at 2 weeks after induction. Represented as percentage of total mammary gland area. (**L**) FACS sorting for PyMT^+^ tumor cells in the blood of WT MIC mice treated with anti-Chi3l1 neutralizing antibody or IgG2b isotype control at 2 weeks after induction. (**M**) Quantification of PyMT^+^ cells as percentage of total live cells in the blood of WT MIC mice treated with IgG2b (*n* = 5) or anti-Chi3l1 (*n* = 5) for 2 weeks. *P < 0.05, **P < 0.01, ***P < 0.001, ****P < 0.0001 by unpaired Student’s *t* test. Scale bars: 1 mm in **B**, left panels; 100 μm in **B**, right panels; 10 μm in **F** and **I**.

As our experiments were done in the CHI3L1 overexpression context, we sought to complement these studies using a germline CHI3L1-deficient mouse model (*Chi3l1^–/–^*) (21) (Figure 6A). These mice were crossed with our MIC GEMM, and lungs were collected at mammary tumor endpoint to assess metastatic spread. Histological analyses revealed that CHI3L1-deficient lungs exhibited a marked reduction in metastatic content (Figure 6, B–E). While 80% of WT mice exhibited lung metastases, only 25% of *Chi3l1^–/–^* mice had metastatic lesions (Figure 6, B and C). Furthermore, the area and number of metastatic lesions were significantly reduced in CHI3L1-deficient mice (Figure 6, B–E).

Immunofluorescent staining of WT and CHI3L1-deficient mammary glands further demonstrated a significant reduction in neutrophil levels upon CHI3L1 ablation (Figure 6, F–H). Moreover, CHI3L1-deficient mammary glands exhibited marked retention of the ECM components laminin and collagen IV (Figure 6, I–K). Overall, these data complement our observations in CHI3L1 OE mice and further support a model in which CHI3L1-driven neutrophil recruitment promotes ECM degradation and thereby fuels metastatic dissemination.

To assess whether therapeutic targeting of CHI3L1 can block this process, we treated STAT3-proficient MIC mice with a neutralizing anti-CHI3L1 antibody (32). Immunofluorescent staining revealed that anti-CHI3L1–treated glands exhibited a marked reduction in neutrophil infiltration, which correlated with increased ECM integrity (Supplemental Figure 12, A–F). Moreover, CHI3L1 neutralization significantly reduced PyMT^+^ CTCs (Figure 6, L and M). Taken together, these findings identify the CHI3L1-neutrophil axis as a critical driver of metastasis that can be therapeutically targeted.

**Figure 7:**
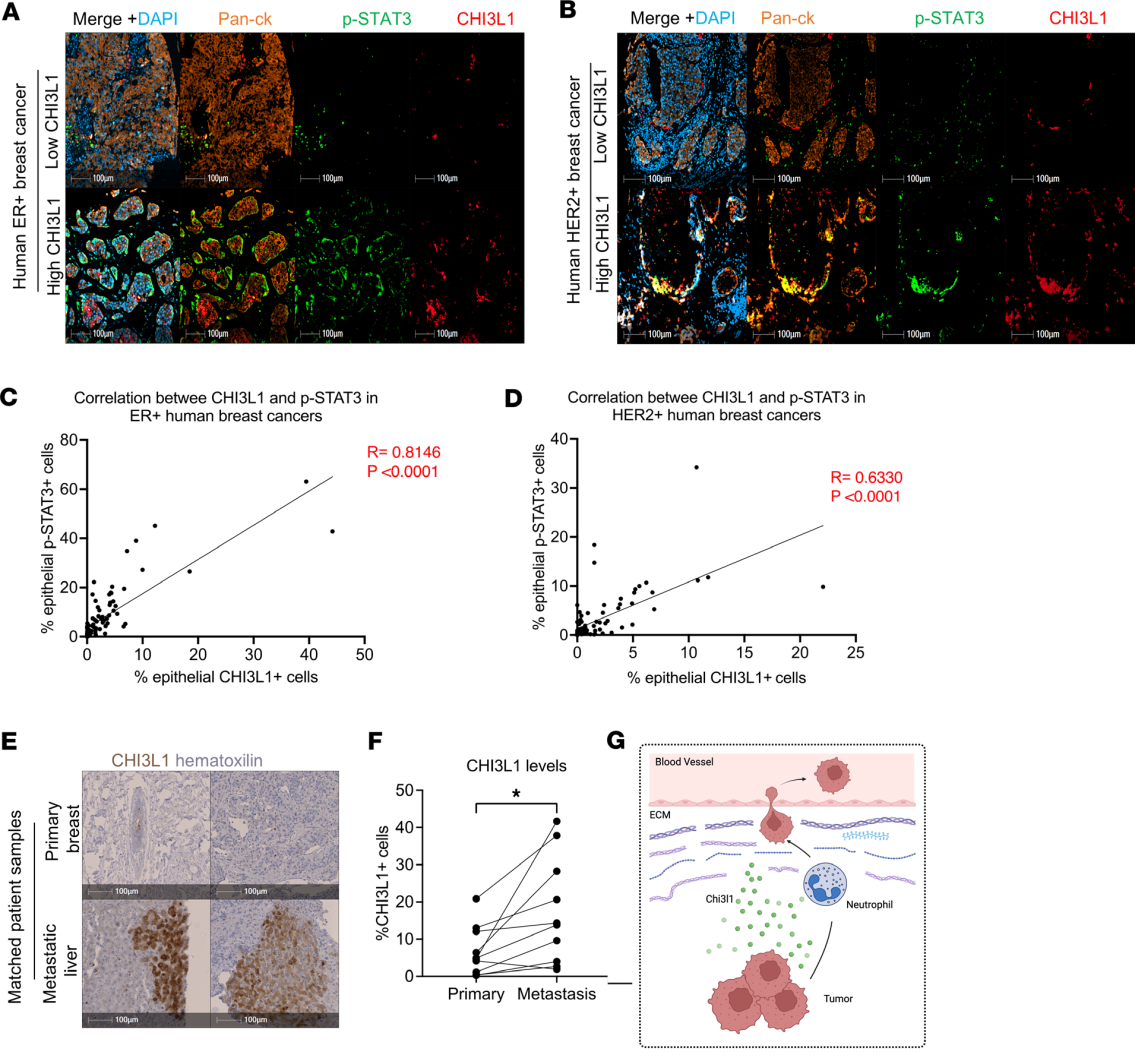
CHI3L1 correlates with STAT3 and is enriched in metastatic patient tumors. (**A** and **B**) Staining of human ER^+^ or HER2^+^ breast cancer samples for CHI3L1, p-STAT3, pan-CK, and DAPI. Micrograph represents areas of high CHI3L1 and low CHI3L1 from different patient samples. (**C** and **D**) Pearson’s correlation between levels of epithelial CHI3L1 and epithelial p-STAT3 in human ER^+^ (*n* = 89) and HER2^+^ (*n* = 85) breast cancer patient samples. (**E**) IHC staining of human primary breast tumor and matched metastatic samples, for CHI3L1, counterstained with hematoxylin. Side-by-side pictures are derived from the same patient. (**F**) Quantification of CHI3L1^+^ cells in patient-matched primary breast tumors (*n* = 10) and recurrent metastatic tumors (*n* = 10). (**G**) Summary of the proposed model for Chi3l1-mediated cancer cell dissemination. Cancer cells secrete Chi3l1 that recruits neutrophils, which degrade the ECM and enhance CTCs. Created in BioRender (Muller W, 2026, https://BioRender.com/kk5b3ud). *P < 0.05, paired Student’s *t* test. Scale bars: 100 μm.

Our previous work demonstrated a positive correlation between CHI3L1 and STAT3 in triple-negative breast cancer patient samples (21). To assess this relationship in other breast cancer subtypes, we stained for CHI3L1 and p-STAT3 in ER^+^ and HER2^+^ human breast cancer tissue microarrays. We observed a strong positive correlation between CHI3L1 and p-STAT3 (Figure 7, A–D), arguing that this targetable axis may be important across all breast cancer subtypes. To further evaluate the role of CHI3L1 in breast cancer metastasis, we stained for CHI3L1 in patient-matched primary breast and metastatic tumors (33). Our results revealed that CHI3L1 was significantly enriched in metastatic tumors compared with the matched primary breast tumors (Figure 7, E and F). These clinical data support our preclinical GEMM studies and demonstrate the critical role that CHI3L1 plays in metastasis. They also highlight CHI3L1 as a potential biomarker for disease progression and metastatic dissemination.

## Discussion

Immunosuppression and metastatic dissemination are hallmarks of cancer progression and major contributors to poor patient outcomes (34–36). This study demonstrates that ectopic overexpression of CHI3L1 recruits neutrophils that generate an immunosuppressive TIME, accelerate mammary tumorigenesis, and promote metastasis. STAT3 ablation in the MIC GEMM delays mammary tumor onset and inhibits lung metastasis (12). Remarkably, elevated CHI3L1 expression completely reversed the tumorigenic defects observed in STAT3-deficient mice, underscoring CHI3L1 as a critical mediator of STAT3-driven cancer progression. Furthermore, genetic ablation or therapeutic inhibition of CHI3L1 inhibited metastatic dissemination. This positions CHI3L1 as a promising therapeutic target for STAT3^+^ breast cancer patients, especially given the challenges of directly targeting STAT3 in the clinic (37). Several CHI3L1-targeting therapies have been used in animal models, including neutralizing antibodies and inhibitors (21, 32, 38). However, these agents have yet to be tested in clinical trials.

Metastasis is the most severe diagnostic indicator for breast cancer patients and the leading cause of cancer-related deaths worldwide (1, 39). Despite its significance, there is a critical lack of clinical biomarkers to predict metastasis and effective therapies to prevent or treat it (3). Multiple studies have linked CHI3L1 to disease aggressiveness and metastasis in animal models (20, 40–42). However, the underlying mechanism through which CHI3L1 promotes metastasis remained unclear. In this study, we provide mechanistic evidence that CHI3L1 relies on neutrophils to degrade the ECM and facilitate metastatic dissemination.

Neutrophils are major contributors to cancer metastasis, through their ability to modify the ECM and suppress antitumor immune responses as well as supporting cancer cells in transit to the site of metastasis (43–46). Elevated levels of neutrophils in breast cancer samples are associated with poor patient outcomes and poor response to therapies (47, 48). Our data indicate that both NETosis and degranulation are involved in the erosion of the ECM and metastatic spread. NETosis-associated proteases and neutrophil-secreted granules both include NE, MMP-9, and cathepsin G, all of which can directly cleave laminin, collagen, and other ECM proteins (49, 50). Identifying the exact neutrophil-secreted products that contribute to ECM remodeling and cancer cell dissemination requires additional investigation.

While studies have shown that targeting neutrophils effectively inhibits metastasis, identifying tumor-derived cytokines that regulate neutrophil-mediated metastasis has been challenging (51). In this regard, we demonstrate that CHI3L1 acts on neutrophils to remodel the ECM and promote metastatic dissemination (Figure 7G). Given the difficulty of directly targeting neutrophils in cancer patients, the ability to inhibit their pro-metastatic functions by targeting tumor-derived cytokines, like CHI3L1, holds significant clinical promise.

Overall, our findings demonstrate that CHI3L1 utilizes neutrophils to establish an immunosuppressive TIME that accelerates mammary tumor onset. Furthermore, CHI3L1 recruits neutrophils that remodel the ECM and promote cancer cell dissemination. Thus, targeting CHI3L1 offers a promising therapeutic strategy to modulate the TIME, suppress tumor growth, and inhibit metastasis, addressing a critical need in breast cancer treatment.

## Methods

### Sex as a biological variable.

Breast cancer is the most common cancer affecting women worldwide (52). Less than 1% of cases occur among men (53). For this reason, we use female GEMMs in our research to accurately reflect the epidemiology of the disease. Because male breast cancer cases are rare, our human tissue sections were all derived from female patients.

### Mouse models.

All procedures described in this study were performed according to the guidelines of the Canadian Council on Animal Care and approved by the Animal Care Committee of McGill University (Animal Use Protocol MCGL5518). All animals were maintained in the Goodman Cancer Institute’s specific pathogen–free facility. The MTB, MIC, conditional *Stat3^–/–^*, and germline *Chi3l1^–/–^* strains have been characterized previously (10, 12, 21, 54, 55). The MTB strain was generously provided by Lewis Chodosh (University of Pennsylvania, Philadelphia, Pennsylvania, USA) (54). The *Stat3^–/–^* strain was provided by David Levy (New York University, New York, New York, USA) (55). Doxycycline induction of MTB MIC mice leads to expression of the PyMT oncogene and Cre recombinase in the mammary epithelium (11) (Figure 1A). Cre excises the conditional *Stat3* alleles, leading to the formation of mammary tumors that are STAT3 deficient.

In order to generate the CHI3L1 OE (CHI3L1-IRES-Cre) GEMM, the TetO-Chi3l1-IRES-Cre plasmid was microinjected into FVB embryos at the one-cell stage. The plasmid was generated by cloning of the different elements into a pMiniT plasmid (New England Biolabs E1203S). The plasmid was diluted out in TE Low EDTA buffer (Thermo Fisher Scientific J75793.AP) at a final concentration of 3 ng/μL and delivered directly into the female or male pronucleus of fertilized FVB embryos. Microinjected embryos were then transferred into CD-1 surrogate female mice to deliver live pups, which were screened for the TetO-Chi3l1-IRES-Cre insert. Successful overexpression of CHI3L1 was confirmed through genotyping (Table 1), qPCR, immunoblots, and RNA FISH.

All animals were maintained on a uniform FVB/N background. Experimental female virgin animals were induced with doxycycline (2 mg/mL; Wisent) in their drinking water at 8–12 weeks of age. Mice on doxycycline were monitored for tumors through weekly physical palpations and caliper measurements. Ethical humane endpoint was defined by the animal care guidelines as a single tumor of 2.5 cm^3^ or multiple tumors per mouse totaling 6.0 cm^3^.

### Pharmacological treatments.

To deplete neutrophils, cohorts of MTB MIC CHI3L1 OE mice were randomized to receive 400 μg of purified anti-LY6G rat antibody (1A8, Bio X Cell BE0075-1) or 400 μg of IgG2a rat isotype control (2A3, Bio X Cell BE0089) via intraperitoneal (i.p.) injections twice weekly. Injections began 5 days before doxycycline induction and continued for 2 weeks after induction (Supplemental Figure 7A). The procedure was repeated to deplete neutrophils in MTB MIC *Stat3^–/–^* CHI3L1 OE mice.

To deplete CD8^+^ T cells, WT MTB MIC mice were randomly divided to receive 500 μg of anti-CD8α rat antibody (YTS 169.4, Bio X Cell BP0117) or 500 μg of rat IgG2b isotype control (LTF-2, Bio X Cell BE0090) via i.p. injections every 3 days. Injections began 5 days before doxycycline induction and continued for 2 weeks after induction. Both neutrophil- and CD8^+^ T cell–depleted mice were subjected to a mandibular bleed at 1 week after induction as well as cardiac puncture at 2 weeks after induction to confirm the depletion in the blood. Depletion was also verified through mammary gland staining, confirming successful depletion that persisted throughout the experiment.

In order to inhibit NETosis or neutrophil degranulation, cohorts of MTB MIC CHI3L1 OE mice were randomized to receive 20 mg/kg GSK484 (Pad4i; MedChemExpress catalog HY-100514) or 30 mg/kg Nexinhib20 (Nex20; HY-125792, MedChemExpress) via i.p. injections as previously described (29, 30, 56–58). Both agents were dissolved in PBS, DMSO, polyethylene glycol, and Tween 80. Treatment took place daily starting 5 days before doxycycline induction and continued for 2 or 6 weeks after induction. Control mice were given mock (vehicle) injections at the same time and for the same duration. Immunofluorescent staining, immunoblot, and ELISA analyses validated successful inhibition of NETosis or degranulation without impacting overall neutrophil recruitment (Supplemental Figure 9).

For anti-CHI3L1 treatments, WT MTB MIC mice were randomly divided to receive 200 μg of anti-CHI3L1 (32) neutralizing antibody through i.p. injections. The injections took place every other day, starting 5 days before induction and continuing for 2 weeks while on doxycycline. Control mice received 200 μg of IgG2b (MCP-11, Bio X Cell BE0086l) isotype control on the same days and for the same duration of time. Anti-CHI3L1 antibody was generated and provided by Jack Elias (Brown University) (32).

### Human subjects.

Patient matched primary breast tumors and metastatic samples have been described previously (33). Briefly, all samples were collected after informed written patient consent and with IRB approval (Dana-Farber/Harvard Cancer Center protocols 09-204 and 05-246). All samples were from treatment-naive female patients and were clinically documented to be ER^+^HER2^–^.

Human ER^+^ (BR1507 and BR1508) and HER^+^ (BR729 and BR1506) breast cancer samples were purchased from TissueArray.com (formerly US Biomax Inc.). Patient information and diagnoses are available at https://www.tissuearray.com

### RNA FISH.

RNAscope in situ hybridization was performed on paraffin-embedded mammary gland sections using RNAscope 2.5 HD Assay-RED kit (Advanced Cell Diagnostics, 322360) according to the manufacturer’s protocol. The following probes were used: mouse *Chi3l1* (449621) and mouse *IFNG* (311391). This protocol was followed with fluorescent IHC as described below.

### RNA extraction and RT-qPCR.

Total RNA was extracted from flash-frozen mammary glands using the Monarch Spin RNA Isolation kit (mini) (New England Biolabs T2110S). For lung qPCR analyses, RNA was extracted from FFPE tissue sections of metastatic lungs sampled at the mouse tumor burden endpoint. Tissue slides were scraped off with a razor blade into an Eppendorf tube and deparaffinized using xylene. The samples were washed with ethanol, then dried, and RNA was extracted using the same kit as above. cDNA was then prepared using the Transcript All-in-One First-Strand cDNA Synthesis SuperMix (TransGen Biotech AT341-02). Real-time RT-qPCR was done on a LightCycler 480 instrument (Roche) using LightCycler 480 SYBR Green 1 Master Mix (Roche 04887352001) and analyzed using the associated software. Samples were run in duplicates and normalized to *β-actin* as control. The primer sequences are listed in Table 1.

### Protein extraction and immunoblots.

Flash-frozen mammary gland pieces at 2 weeks after induction were crushed using a mortar and a pestle under liquid nitrogen. Samples were lysed using RIPA buffer (10 mM Tris at pH 8, 1 mM EDTA, 0.5 mM EGTA, 1% Triton X-100, 0.1% sodium deoxycholate, 0.1% SDS, 140 mM NaCl) with protease inhibitor cocktail (1 μg/mL aprotinin, 1 μg/mL leupeptin, 1 mM sodium orthovanadate, and 1 μg/mL phenylmethylsulfonyl fluoride) and left to rotate at 4°C for 60 minutes. Lysates were centrifuged at 14,000*g* for 10 minutes at 4°C, and the supernatant was collected.

Immunoblot was performed as previously described (59, 60) using the following antibodies: mouse CHI3L1 (1:1,000; Invitrogen PA5-8135), STAT3 (1:1,000; Cell Signaling 9139), p-STAT3 (1:1,000; Cell Signaling 9145), CitH3 (1:1,000; Abcam Ab5103), vinculin (1:2,000; Millipore MAB3574), and α-tubulin (1:1,000; Cell Signaling 3873). The Odyssey CL-C imaging system was used to image the membranes. Images were analyzed using Image Studio software (LICOR Biosciences) where each protein target was normalized to loading control on the same membrane.

### ChIP.

MMTV-PyMT STAT3-proficient and -deficient cell lines were generated by CRISPR and described previously (61). STAT3-deficient cells were provided by Josie Ursini-Siegel (McGill University). Cells were grown to 90% confluence in 15 cm plates. ChIP was then performed using the SimpleChIP Enzymatic Chromatin IP Kit (Cell Signaling 9003) according to the manufacturer’s protocol. The following antibodies were used: STAT3 (1:50; Cell Signaling 9139), H3 (1:50; Cell Signaling 4620), and rabbit IgG (1:50; Cell Signaling 2729). After precipitation, qPCR was performed on the precipitated DNA (Table 1). qPCR results were normalized to H3 precipitation for each primer. The experiment was performed on 2 separate biological cell lines and was repeated twice.

### Multiplex fluorescent IHC.

FFPE tissue sections were stained as previously described (21, 62). Target retrieval was performed in citrate buffer (pH 6; Vector Laboratories H-3300-250) or EDTA buffer (pH 9; Vector Laboratories H-3301-250), and blocking was done with casein-based buffer (Vector Laboratories SP-5020). After primary antibody, ImmPRESS HRP Polymer secondary antibody (Vector Laboratories; anti-mouse VECTMP745250 or anti-rabbit VECTMP740150) was added. For fluorescent staining, Opal working solution containing tyramide signal amplification substrates (OPAL 520: OP-001001; OPAL 570: OP-001003; OPAL 620: OP-001004; or OPAL 690: OP-001006; Akoya Biosciences) was used. Samples were counterstained with DAPI and mounted for imaging. For traditional IHC, the same procedure was repeated, but the following substrate was used: 3,30-diaminobenzidine (DAB) (Cell Signaling 8059). In this case, slides were counterstained with hematoxylin for 30 seconds.

Sections were scanned using the AxioScan Z1 digital slide scanner (Carl Zeiss). HALO (Indica Lab) was used for analyses using the following algorithms: Multiplex IHC v2.3.4 and Area Quantification FL v3.0.1. Tumor infiltration analysis was performed using HALO’s classifier function. The entire tissue section was analyzed for all staining experiments to avoid sampling bias. All quantification is represented as percentage of all cells unless otherwise specified.

For ECM analyses, staining positivity was quantitatively defined using HALO’s Area Quantification FL v3.0.1 algorithm. This teachable algorithm was trained to recognize the fluorophore-specific signal for each ECM component and to calculate area fraction (positive staining as a percentage of total tissue area). Threshold parameters were standardized during algorithm training and then kept constant across all samples to ensure consistency. Supplemental Figure 12G is an example of the thresholding for each stain. All analyses were performed in a blinded manner, and all samples were analyzed using the same batch settings.

The following primary antibodies were used: CD3 (1:200; Abcam ab16669), CD8 (1:200; Cell Signaling 98941), CD4 (1:50; Cell Signaling 25229), PD-1 (1:200; Cell Signaling 84651), granzyme B (1:200; Cell Signaling 44153), p-STAT3 (1:50; Cell Signaling 9145), Ly6G (1:100; Cell Signaling 87048), NE (1:200; Cell Signaling 90120), MPO (1:500; Abcam ab208670), F4/80 (1:400; Cell Signaling 70076), CD206 (1:400; Cell Signaling 24595), p-STAT1 (1:400; Cell Signaling 9167), laminin (1:200; Abcam ab11575), collagen IV (1:100; Abcam ab6586), pan-cytokeratin (pan-CK) (1:10; Ventana 760-2595), and CHI3L1 (1:400; Cell Signaling 47066). All antibodies were diluted in 2% BSA TBST.

### FACS analysis.

FACS analysis on blood or mammary gland tissue was performed as previously described (21). For CTC analyses, PyMT^+^ cells were gated out of live cells in the blood. The following antibodies were used: Viability Dye eFluor 506 (Thermo Fisher Scientific/eBioscience 65-0866-14), CD45 (BD Biosciences 564225), CD3 (BioLegend 100204), CD8 (BioLegend 100747), CD4 (BioLegend 100422), PD-1 (BioLegend 135221), CD44 (BioLegend 103008), F4/80 (BioLegend 123114), Ly6c (BioLegend 128046), CD11b (BioLegend 101201), CD11c (BioLegend 117327), Ly6G (BioLegend 127606), CD206 (BioLegend 141706), CD86 (BioLegend 105032), and PyMT (Santa Cruz Biotechnology sc-53481). Flow cytometry was done in the BD LSR Fortessa 4-laser (405/488/561/633 nm) flow cytometer, and the results were analyzed using FlowJo 10.6.2 (Tree Star).

### Enzyme-linked immunosorbent assay.

Blood was collected from Nex20- or vehicle-treated CHI3L1 OE MIC mice at 2 and 6 weeks after induction through cardiac puncture. The blood was allowed to clot for 1 hour at room temperature before centrifugation for 20 minutes at 1,000*g* at 4°C. ELISA for serum NE (R&D Systems catalog DY4517-05) or MPO (R&D Systems catalog DY3667) was performed according to the manufacturer protocol at 1 in 50 dilution. Each experiment was done in duplicates and repeated twice.

### Primary cell culture and cell lines.

The PyMT^+^ cell line was established from primary mammary tumors from MMTV-PyMT transgenic mice as described previously (63). Cells were maintained in DMEM (Wisent 219-010) medium supplemented with 10% FBS, 5 ng/mL EGF, 35 μg/mL bovine pituitary extract, 5 μg/mL insulin, 1 μg/mL hydrocortisone. Extraction of primary mouse low-density neutrophils (LDNs) from peripheral blood of tumor-bearing mice has been described previously (64). LDNs were cultured in RPMI medium (Wisent 350-000-CL) supplemented with 3% FBS, 5% penicillin/streptomycin, and 5% amphotericin. All cells were incubated at 37°C, 5% CO_2_ conditions.

### Transwell invasion assay.

Transwell insert polyethylene terephthalate membranes with 8.0 mm pore size were coated in 100 μL of synthetic ECM Cultrex Growth Factor Basement Membrane Extract (R&D Systems catalog 3433-010-01) diluted with sterile water at a 1 in 2 concentration. A 24-well plate containing the Transwell inserts with Cultrex was placed for 1 hour at 37°C and 5% CO_2_ to allow Cultrex to solidify as described previously (65). PyMT^+^ cells were seeded at 1.5 × 10^5^ cells in 3% RPMI onto the Cultrex. The 3% RPMI medium was supplemented with PBS, 200 ng/mL of rmChi3l1 (R&D Systems, catalog 2649-CH), and/or 5 × 10^5^ LDNs (Supplemental Figure 4E). The Transwell inserts were then placed in the 24-well plate containing RPMI medium supplemented with 10% FBS. Each condition was run in triplicates, and the experiment was repeated 3 times. After 12 hours of incubation at 37°C, 5% CO_2_, the filters were removed. The cells that invaded through the membrane were fixed in 10% neutral-buffered formalin for 30 minutes, and filters were counterstained with crystal violet. Image acquisition of stained cells was performed with an EVOS M7000 Invitrogen microscope (objective ×10). Cells were manually counted on HALO.

This assay was repeated using conditioned medium derived from murine LDNs treated with vehicle, Nex20 to inhibit degranulation, or Pad4i to inhibit NETosis. LDNs were maintained in 2 mL of 3% FBS RPMI for 10 hours. Control neutrophils received vehicle (10% DMSO, 75% PBS, 15% Tween 80). To inhibit degranulation, a second population of neutrophils received 30 μM of Nexinhib20 (MedChemExpress catalog HY-125792). To inhibit NETosis, a third population of neutrophils received 300 μM of GSK484 hydrochloride (Pad4i; MedChemExpress catalog HY-100514). Plates were kept at 37°C and 5% CO_2_. Conditioned medium was then centrifugated, aliquoted, and kept at –80°C until use.

### In vitro laminin and collagen degradation assay.

A 96-well glass-bottom microscopy plate (Thermo Fisher Scientific, Nunc 165305) was coated with Cultrex Growth Factor Basement Membrane Extract (R&D Systems catalog 3433-010-01). The plate was incubated at 37°C in 5% CO_2_ for 30 minutes to solidify. PyMT^+^ cells were plated at 1 × 10^4^ cells per well in 3% RPMI medium alone or with 2 × 10^5^ murine neutrophils. The plates were incubated for 12 hours. Cells were then fixed with 2% paraformaldehyde and permeabilized with PBS containing 0.5% Triton X-100 for 10 minutes at room temperature, followed by blocking using PBS with 0.2% Triton X-100 and 0.05% Tween 20 (IF buffer) and 2% BSA for 30 minutes at room temperature. Primary anti-laminin antibody (1:200; Abcam Ab11575) or Ki67 (1:100; Cell Signaling 12202) was added and incubated overnight at 4°C. For actin staining, Alexa Fluor Plus 555 phalloidin was used (1:500; Invitrogen A30106). Goat anti-rabbit secondary antibody Alexa Fluor 555 (Invitrogen A21428) was added for 40 minutes at room temperature. The cells were then counterstained with DAPI.

For EdU (Thermo Fisher Scientific A10044) incorporation and staining, antibody stock was added directly to the cells and mixed with the medium 2 hours before endpoint at a 10 μM final concentration. After fixing, permeabilization, and blocking as described above, detection of Edu-DNA was performed with a homemade kit similar to the Invitrogen Click-iT EdU Alexa Fluor 488 flow cytometry kit with copper II sulfate CuSO_4_ (Aldon), CF405M Dye Azide (Biotium), and sodium ascorbate (Bioshop).

For the collagen degradation assay, a 96-well plate was coated with DQ collagen, type IV from human placenta, fluorescein conjugate (Invitrogen D12052). DQ collagen was prepared at a 1 mg/mL concentration in distilled water. The stock was then diluted to a working concentration of 25 μg/mL in 0.1% unconjugated gelatin. PyMT^+^ cancer cells with or without neutrophils were added as described above and were incubated at 37°C in 5% CO_2_ for 12 hours. The cells were then stained with Alexa Fluor Plus 555 phalloidin and counterstained with DAPI. At this point, the degraded collagen would appear as green fluorescence. All Transwell invasion assays and laminin and collagen IV degradation assays were performed in triplicates and repeated twice.

All assays were imaged using a Zeiss AxioObserver fully motorized inverted confocal microscope LSM 710 with a ×10 objective and analyzed on HALO.

### IncuCyte cell proliferation assay.

LDN conditioned medium was collected as described above. Twenty thousand PyMT^+^ cells per well were seeded in triplicates in a 24-well plate (Nunc 142475). Either 700 μL of neutrophil conditioned medium or control medium was added to the PyMT^+^ cells. Live cell imaging was performed using the IncuCyte S3 system (ESSEN BioSciences) at ×10 magnification every 4 hours over a period of 4 days (4 images per well per time point). Percentage confluence was determined using IncuCyte S3 Analysis software (v2019A, ESSEN BioSciences).

### Statistics.

All figures and statistical analyses were performed using Prism software (GraphPad). One-way ANOVA with Tukey’s or Dunnett’s post hoc test for multiple comparisons was performed as appropriate. A 2-tailed paired or unpaired Student’s *t* test was used to compare 2 groups as appropriate. Kaplan-Meier onset curve was generated using Prism software and analyzed using log-rank test. Data are plotted as average ± SEM. Each data point is an independent biological sample. The results of the statistical tests can be found within the figures and figure legends. *P* values less than 0.05 were considered statistically significant. Sample size (*n*) is indicated in the figure legends.

### Study approval.

All in vivo experimental endpoints followed guidelines of the Animal Ethics Committee, Facility Animal Care Committee, and Canadian Council on Animal Care (McGill University, Animal Use Protocol MCGL5518). In vivo treatment experimental endpoints were based on previous in vivo experimental experience, published data with respective drugs, and drug optimization pilot studies. No experiments with multiple endpoints were included in this study. This study did not involve a controlled clinical trial or survey. Patient-matched primary breast tumor and recurrent metastatic tumor samples were obtained with IRB approval (Dana-Farber/Harvard Cancer Center protocols 09-204 and 05-246) and with informed written patient consent. We also obtained tissue microarray from ER^+^ (TissueArray.com BR1507 and BR1508) and HER^+^ breast cancer primary tumors (BR729 and BR1506).

### Data availability.

The data generated in this study are available within the article and its supplemental data files. Values for all data points in graphs are reported in the Supporting Data Values file. No sequencing data were generated in this study. This paper does not report original code. Any additional reagents or mouse models used in this paper are available from the corresponding author upon request after a completed material transfer agreement.

## Author contributions

TT, AM, and WJM conceptualized the study. TT, AM, YG, VSG, DZ, ES, YS, HP, SSA, VP, and BX performed investigation. TT, AM, and VSG performed analysis. NUL, MEH, KS, CGL, SK, JUS, JAE, PMS, and RJ provided resources. TT, AM, and WJM wrote the original draft of the manuscript. TT, AM, YG, VSG, DZ, ES, YS, HP, SSA, NUL, MEH, KS, CGL, SK, JUS, JAE, PMS, RJ, and WJM reviewed and edited the manuscript. WJM supervised the study and acquired funding. The order of co–first authors was assigned by flipping a coin.

## Funding support

- Doctoral Vanier Canada Graduate Scholarship provided by the Canadian Institutes of Health Research (CIHR) (funding reference no. CGV 192738) (to TT).
- Donner Foundation Studentship Award awarded through the Goodman Cancer Institute, McGill University (to AM).
- Rolande and Marcel Gosselin Graduate Studentships and Carole Epstein Fellowship in Health, both awarded by the Faculty of Medicine, McGill University (to AM).
- Canada Graduate Scholarship Doctoral Award provided by the CIHR (to YG).
- Doctoral Research Award from the Cancer Research Society (to ES).
- Goodman Cancer Institute Entry Scholarship (to YS).
- CIHR Frederick Banting and Charles Best Canada Graduate Scholarship Master’s (to YS).
- Fonds de Recherche du Québec–Santé Doctoral Training Scholarships (to YS).
- CIHR (PJT-203842) (to JUS).
- Canada Research Chair of Molecular Oncology (to WJM).
- Terry Fox Research Institute Program Group Grant (to PMS and WJM).
- CIHR (Foundation Grant 148373, bridge grant PLL-185690, and Project Grant PJT-190027).
- Canadian Cancer Society Impact Grant (grant 263780).
- Cancer Research Society (grant 1281449).

## Supplementary Material

Supplemental data

Unedited blot and gel images

Supporting data values

## Figures and Tables

**Table 1:**
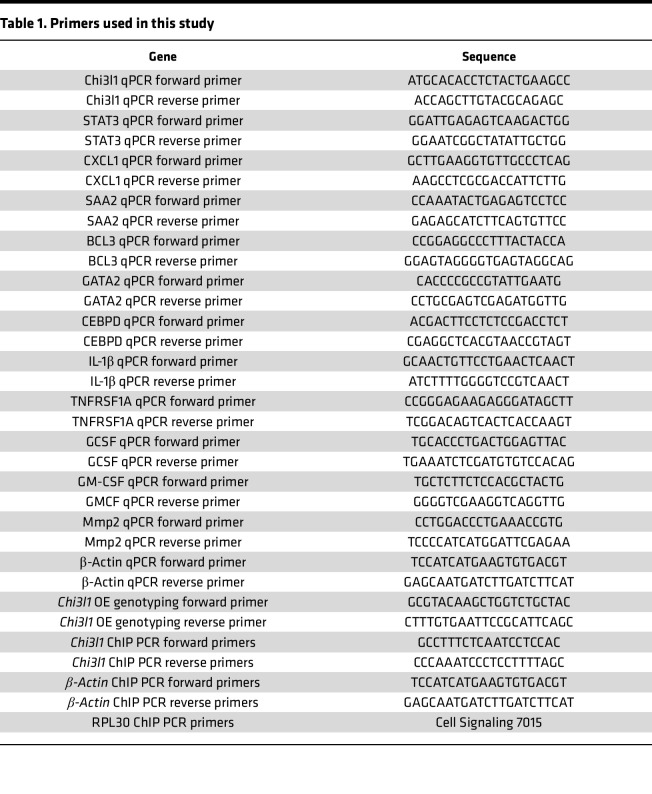
Primers used in this study

## Version 1 02/03/2026

In-Press Preview

## Version 2 03/23/2026

Electronic publication

## References

[1] Scully OJ (2012). Breast cancer metastasis. Cancer Genomics Proteomics.

[2] Kimbung S (2015). Clinical and molecular complexity of breast cancer metastases. Semin Cancer Biol.

[3] Riggio AI (2021). The lingering mysteries of metastatic recurrence in breast cancer. Br J Cancer.

[4] Chauhan SK (2024). Peripheral immune cells in metastatic breast cancer patients display a systemic immunosuppressed signature consistent with chronic inflammation. NPJ Breast Cancer.

[5] Monteran L (2020). Bone metastasis is associated with acquisition of mesenchymal phenotype and immune suppression in a model of spontaneous breast cancer metastasis. Sci Rep.

[6] Liu Y, Cao X (2016). Immunosuppressive cells in tumor immune escape and metastasis. J Mol Med (Berl).

[7] Attalla S (2023). Tailoring therapies to counter the divergent immune landscapes of breast cancer. Front Cell Dev Biol.

[8] Qin JJ (2019). STAT3 as a potential therapeutic target in triple negative breast cancer: a systematic review. J Exp Clin Cancer Res.

[9] Banerjee K, Resat H (2016). Constitutive activation of STAT3 in breast cancer cells: a review. Int J Cancer.

[10] Rao T (2014). Inducible and coupled expression of the polyomavirus middle T antigen and Cre recombinase in transgenic mice: an in vivo model for synthetic viability in mammary tumour progression. Breast Cancer Res.

[11] Attalla S (2021). Insights from transgenic mouse models of PyMT-induced breast cancer: recapitulating human breast cancer progression in vivo. Oncogene.

[12] Jones LM (2016). STAT3 establishes an immunosuppressive microenvironment during the early stages of breast carcinogenesis to promote tumor growth and metastasis. Cancer Res.

[13] Singh SK (2011). A complex of nuclear factor I-X3 and STAT3 regulates astrocyte and glioma migration through the secreted glycoprotein YKL-40. J Biol Chem.

[14] Yan C (2013). Stat3 downstream gene product chitinase 3-like 1 is a potential biomarker of inflammation-induced lung cancer in multiple mouse lung tumor models and humans. PLoS One.

[15] Hughes K (2012). Conditional deletion of Stat3 in mammary epithelium impairs the acute phase response and modulates immune cell numbers during post-lactational regression. J Pathol.

[16] Jensen BV (2003). High levels of serum HER-2/neu and YKL-40 independently reflect aggressiveness of metastatic breast cancer. Clin Cancer Res.

[17] Cohen N (2017). Fibroblasts drive an immunosuppressive and growth-promoting microenvironment in breast cancer via secretion of Chitinase 3-like 1. Oncogene.

[18] Lee CG (2009). Role of breast regression protein 39 (BRP-39)/chitinase 3-like-1 in Th2 and IL-13-induced tissue responses and apoptosis. J Exp Med.

[19] Ma B (2022). CHI3L1 enhances melanoma lung metastasis via regulation of T cell co-stimulators and CTLA-4/B7 axis. Front Immunol.

[20] Geng B (2018). Chitinase 3-like 1-CD44 interaction promotes metastasis and epithelial-to-mesenchymal transition through β-catenin/Erk/Akt signaling in gastric cancer. J Exp Clin Cancer Res.

[21] Taifour T (2023). The tumor-derived cytokine Chi3l1 induces neutrophil extracellular traps that promote T cell exclusion in triple-negative breast cancer. Immunity.

[22] Carpenter RL, Lo HW (2014). STAT3 target genes relevant to human cancers. Cancers (Basel).

[23] Kim EG (2020). Chitinase 3-like 1 contributes to food allergy via M2 macrophage polarization. Allergy Asthma Immunol Res.

[24] Kawada M (2012). Chitinase 3-like 1 promotes macrophage recruitment and angiogenesis in colorectal cancer. Oncogene.

[25] Oskarsson T (2013). Extracellular matrix components in breast cancer progression and metastasis. Breast.

[26] Strøbech JE (2022). Neutrophil granulocytes influence on extracellular matrix in cancer progression. Am J Physiol Cell Physiol.

[27] Rawat K (2021). Neutrophil-derived granule cargoes: paving the way for tumor growth and progression. Cancer Metastasis Rev.

[28] Koenderman L, Vrisekoop N (2025). Neutrophils in cancer: from biology to therapy. Cell Mol Immunol.

[29] Johnson JL (2016). Identification of neutrophil exocytosis inhibitors (Nexinhibs), small molecule inhibitors of neutrophil exocytosis and inflammation: druggability of the small GTPase Rab27a. J Biol Chem.

[30] Lewis HD (2015). Inhibition of PAD4 activity is sufficient to disrupt mouse and human NET formation. Nat Chem Biol.

[31] Tallón de Lara P (2022). Antimetastatic defense by CD8^+^ T cells. Trends Cancer.

[32] Ma B (2021). CHI3L1 regulates PD-L1 and anti-CHI3L1-PD-1 antibody elicits synergistic antitumor responses. J Clin Invest.

[33] Gu Y (2024). Osteopontin is a therapeutic target that drives breast cancer recurrence. Nat Commun.

[34] Hanahan D, Weinberg RA (2011). Hallmarks of cancer: the next generation. Cell.

[35] Ali H (2014). Association between CD8^+^ T-cell infiltration and breast cancer survival in 12,439 patients. Ann Oncol.

[36] Gu Y (2022). Exploiting mouse models to recapitulate clinical tumor dormancy and recurrence in breast cancer. Endocrinology.

[37] Taniguchi K (2021). A brief update on STAT3 signaling: current challenges and future directions in cancer treatment. J Cell Signal.

[38] Lee YS (2022). A small molecule targeting CHI3L1 inhibits lung metastasis by blocking IL-13Rα2-mediated JNK-AP-1 signals. Mol Oncol.

[39] Giaquinto AN (2022). Breast cancer statistics, 2022. CA Cancer J Clin.

[40] Chen Y (2017). Tumor-recruited M2 macrophages promote gastric and breast cancer metastasis via M2 macrophage-secreted CHI3L1 protein. J Hematol Oncol.

[41] Luo D (2017). CHI3L1 overexpression is associated with metastasis and is an indicator of poor prognosis in papillary thyroid carcinoma. Cancer Biomark.

[42] Libreros S (2012). Induction of proinflammatory mediators by CHI3L1 is reduced by chitin treatment: decreased tumor metastasis in a breast cancer model. Int J Cancer.

[43] Xiong S (2021). Neutrophils in cancer carcinogenesis and metastasis. J Hematol Oncol.

[44] Cools-Lartigue J (2013). Neutrophil extracellular traps sequester circulating tumor cells and promote metastasis. J Clin Invest.

[45] Hajizadeh F (2021). Tumor-associated neutrophils as new players in immunosuppressive process of the tumor microenvironment in breast cancer. Life Sci.

[46] Bekes EM (2011). Tumor-recruited neutrophils and neutrophil TIMP-free MMP-9 regulate coordinately the levels of tumor angiogenesis and efficiency of malignant cell intravasation. Am J Pathol.

[47] Zakurdaev EI (2025). The role of tumor-associated neutrophils in early luminal HER2-negative breast cancer progression. Asian Pac J Cancer Prev.

[48] Geng SK (2021). Tumor infiltrating neutrophil might play a major role in predicting the clinical outcome of breast cancer patients treated with neoadjuvant chemotherapy. BMC Cancer.

[49] Albrengues J (2018). Neutrophil extracellular traps produced during inflammation awaken dormant cancer cells in mice. Science.

[50] Zhu Y (2021). Interplay between extracellular matrix and neutrophils in diseases. J Immunol Res.

[51] Wculek SK, Malanchi I (2015). Neutrophils support lung colonization of metastasis-initiating breast cancer cells. Nature.

[52] Fahad Ullah M (2019). Breast cancer: current perspectives on the disease status. Adv Exp Med Biol.

[53] Ly D (2013). An international comparison of male and female breast cancer incidence rates. Int J Cancer.

[54] Gunther EJ (2002). A novel doxycycline-inducible system for the transgenic analysis of mammary gland biology. FASEB J.

[55] Raz R (1999). Essential role of STAT3 for embryonic stem cell pluripotency. Proc Natl Acad Sci U S A.

[56] Mousset A (2023). Neutrophil extracellular traps formed during chemotherapy confer treatment resistance via TGF-β activation. Cancer Cell.

[58] Chen D (2025). DNASE1L3-expressing dendritic cells promote CD8^+^ T cell function and anti-PD-(L)1 therapy efficacy by degrading neutrophil extracellular traps. Cancer Cell.

[59] Xiao B (2020). Rheb1-independent activation of mTORC1 in mammary tumors occurs through activating mutations in mTOR. Cell Rep.

[60] Attalla SS (2023). HER2Δ16 engages ENPP1 to promote an immune-cold microenvironment in breast cancer. Cancer Immunol Res.

[61] Ahn R (2017). The Shc1 adaptor simultaneously balances Stat1 and Stat3 activity to promote breast cancer immune suppression. Nat Commun.

[62] Bui T (2022). Emergence of β1 integrin-deficient breast tumours from dormancy involves both inactivation of p53 and generation of a permissive tumour microenvironment. Oncogene.

[63] Huck L (2010). β1-integrin is dispensable for the induction of ErbB2 mammary tumors but plays a critical role in the metastatic phase of tumor progression. Proc Natl Acad Sci U S A.

[64] Hsu BE (2019). Immature low-density neutrophils exhibit metabolic flexibility that facilitates breast cancer liver metastasis. Cell Rep.

[65] Justus CR (2023). Transwell in vitro cell migration and invasion assays. Methods Mol Biol.

