# Supplementary material for: The CHI3L1-neutrophil axis drives immune suppression and breast cancer metastatic dissemination
Source: JCI Insight. 2026 Feb 3;11(6):e199307. doi: 10.1172/jci.insight.199307 (PMC13043092; doi:10.1172/jci.insight.199307)
Supplement: Supplemental data [file jciinsight-11-199307-s292.pdf]

Title:

The CHI3L1-Neutrophil Axis Drives Immune Suppression and Breast Cancer Metastatic Dissemination

Authors:

Tarek Taifour[1,2,†], Adéline Massé[2,3,†], Yu Gu[2,3], Virginie Sanguin-Gendreau[2], Dongmei Zuo[2], Bin Xiao[2], Emilie Solymoss[1,2], Yunyun Shen[2,3], Hailey Proud[2,3], Sherif Samer Attalla[2], Vasilios Papavasiliou[2], Nancy U. Lin[4], Melissa E. Hughes[4], Kalie Smith[4], Chun Geun Lee[5,6], Suchitra Kamle[5], Josie Ursini-Siegel[1,3,7], Jack A. Elias[5], Peter M. Siegel[1,2,3], Rinath Jeselsohn[4], and William J. Muller*[1,2,3]

Supplemental Information

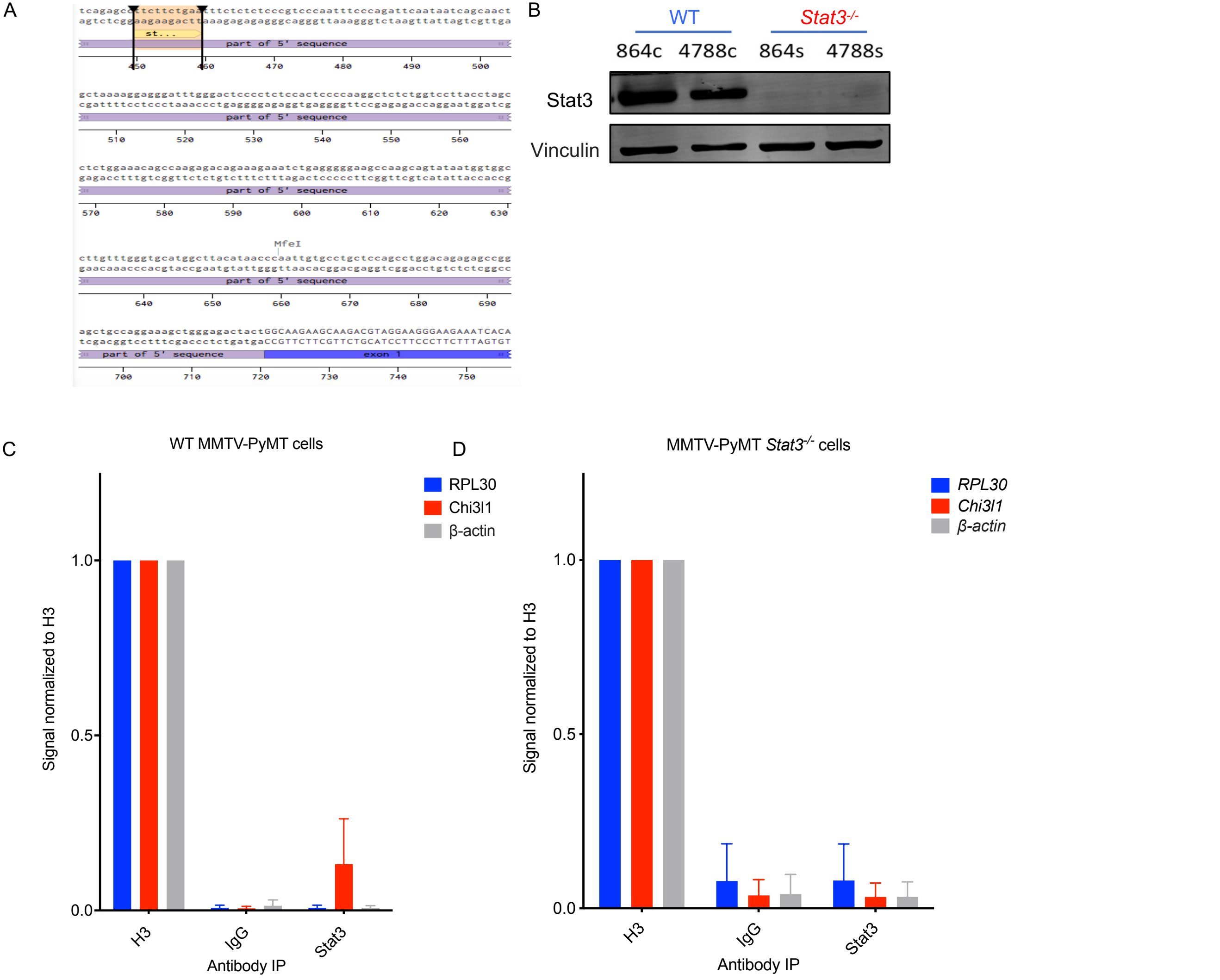

Supplementary Figure 1: Chi3l1 is a direct Stat3 transcriptional target.
(A) Stat3 binding site (yellow) found upstream of the *Chi3l1* gene (dark blue) on mouse chromosome 1. (B) Immunoblot for Stat3 and Vinculin on Stat3 WT (864c, 4788c) and *Stat3*$^{-/-}$ (864s, 4788s) cell lines. (C-D) qPCR quantification of immunoprecipitated cell-lines; (C) WT Stat3 cell lines precipitated with H3, IgG and Stat3 antibodies and qPCR amplified using primers for *RPL30* (blue), *Chi3l1* (red) or *β-actin* (grey). (D) *Stat3*$^{-/-}$ cell lines precipitated with H3, IgG and Stat3 antibodies and qPCR amplified using primers for *RPL30* (blue), *Chi3l1* (red) or *β-actin* (grey). All qPCR data for each primer was normalized to that primer's amplification of H3 precipitated DNA. *n* = 2 biological replicates.

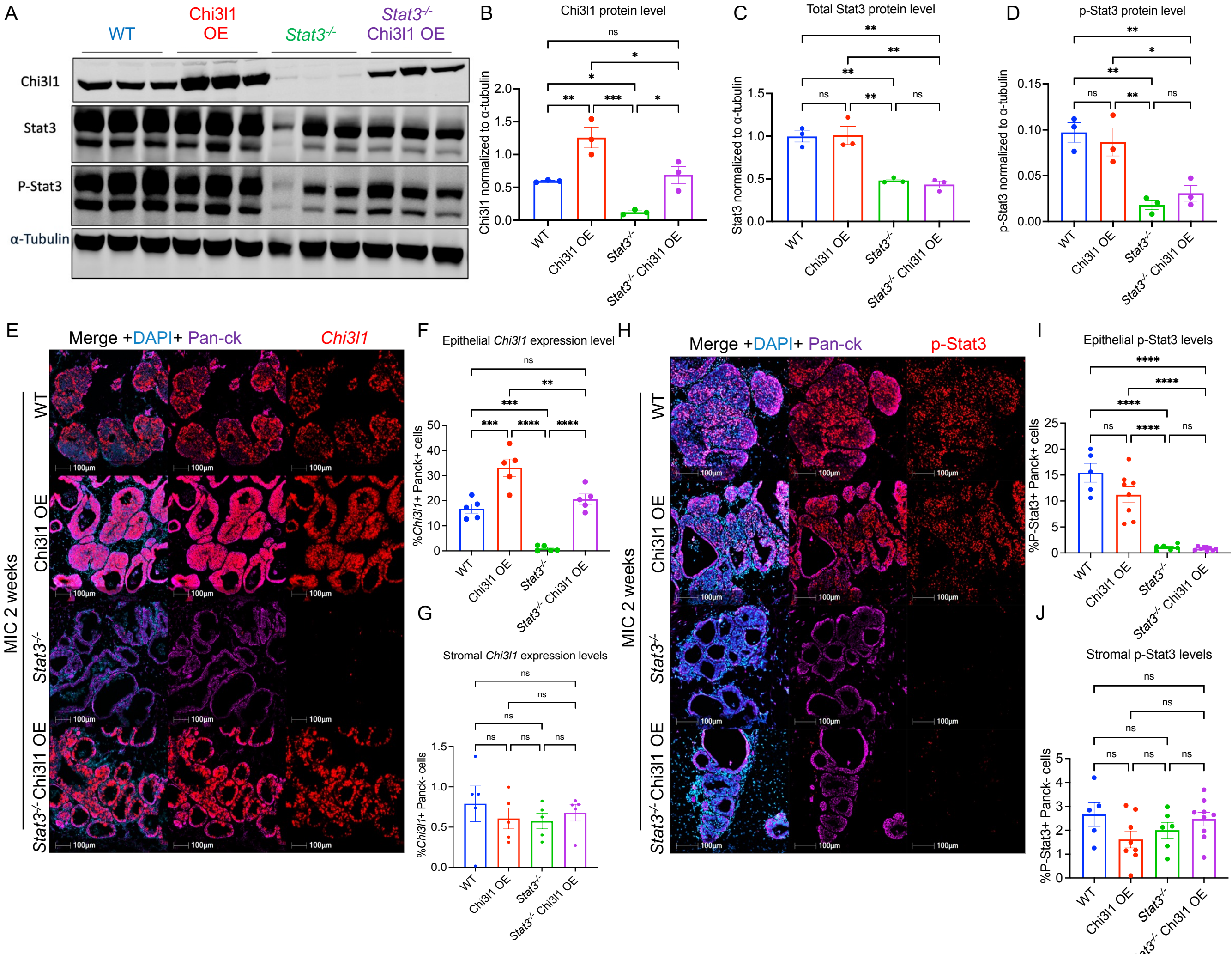

Supplementary figure 2: Validation of Chi3l1 overexpression Stat3-deficient mouse model. (A) Immunoblots for Chi3l1, Stat3, p-Stat3 and α-tubulin on WT (*n* = 3), Chi3l1 OE (*n* = 3), *Stat3*$^{-/-}$ (*n* = 3) and *Stat3*$^{-/-}$ Chi3l1 OE (*n* = 3) MIC mammary glands at 2 weeks post-induction. (B-D) Quantification of Chi3l1, Stat3 and p-Stat3 immunoblots normalized to α-tubulin. (E) RNA FISH staining against *Chi3l1* combined with staining for Pan-ck and DAPI on mammary tissue from WT, Chi3l1 OE, *Stat3*$^{-/-}$ and *Stat3*$^{-/-}$ Chi3l1 OE MIC mammary glands. (F,G) Quantification of *Chi3l1*+Pan-ck+ cells and *Chi3l1*+Pan-ck- cells in WT (*n* = 5), Chi3l1 OE (*n* = 5), *Stat3*$^{-/-}$ (*n* = 5) and *Stat3*$^{-/-}$ Chi3l1 OE (*n* = 5) MIC mammary glands at 2 weeks post induction. (H) Staining of WT, Chi3l1 OE, *Stat3*$^{-/-}$ and *Stat3*$^{-/-}$ Chi3l1 OE MIC mammary glands at 2 weeks post-induction, for p-Stat3 and Pan-ck and DAPI. (I,J) Quantification of p-Stat3+Pan-ck+ cells and p-Stat3-Pan-ck+ cells in WT (*n* = 5), Chi3l1 OE (*n* = 8), *Stat3*$^{-/-}$ (*n* = 6) and *Stat3*$^{-/-}$ Chi3l1 OE (*n* = 9) MIC mammary glands at 2 weeks post induction. Ns: not significant, *$p<0.05$, **$p<0.01$, ***$p<0.001$ and ****$p<0.0001$ by One-Way Anova with Tukey's post-hoc test.

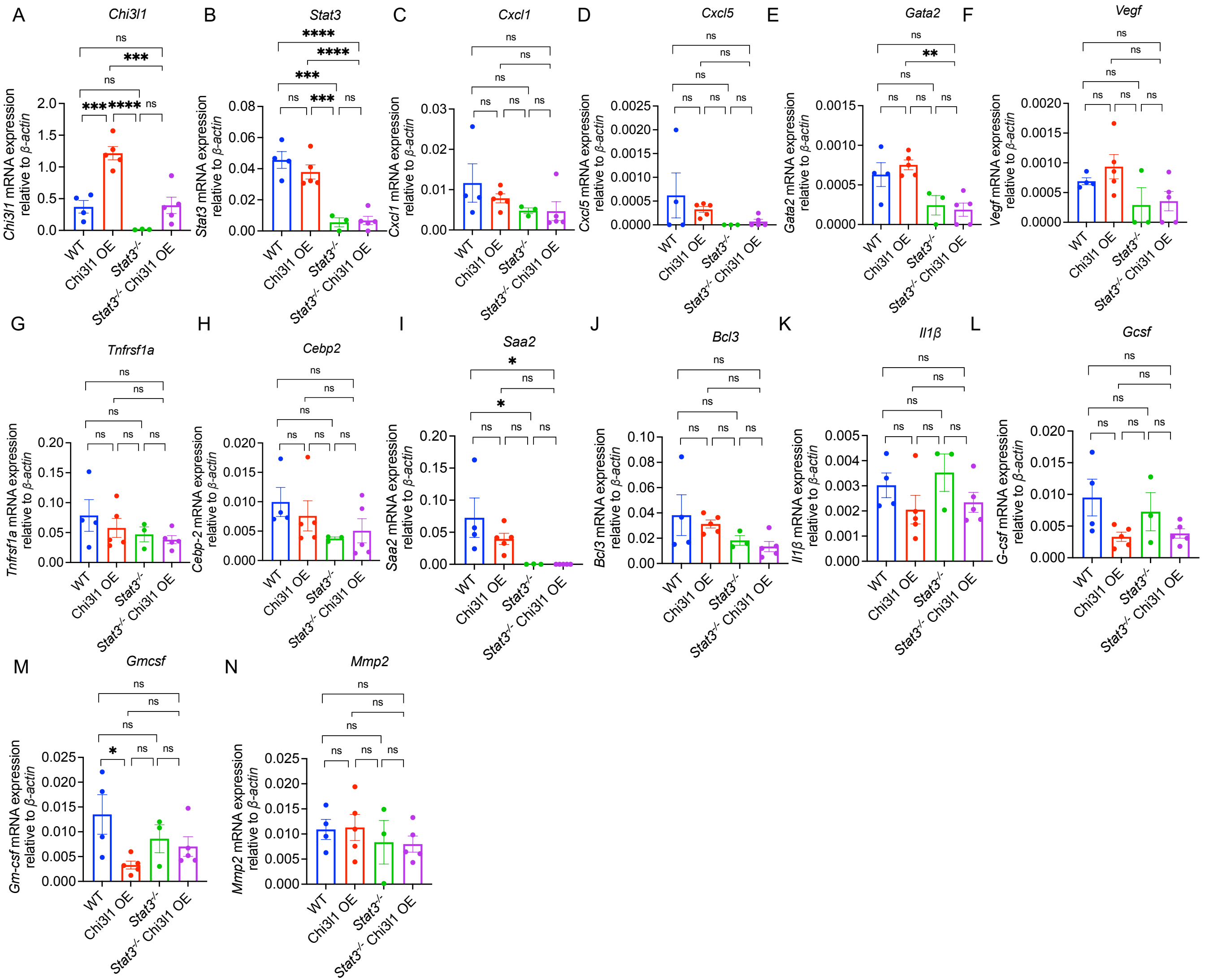

Supplemental Figure 3: Validation of direct Stat3-transcriptional targets.
(A-K) qRT-PCR analysis of *Chi3l1, Stat3, Cxcl1, Cxcl5, Gata2, Vegf, Tnfrsf1a, Cebp-2, Saa2, Bcl3, Il-1*β, *Gcsf, Gm-cf* and *MMP2 l*evels normalized to *β-actin* in WT (*n* = 4) Chi3l1 OE (*n* = 5), *Stat3*$^{-/-}$ (*n* = 3) and *Stat3*$^{-/-}$ Chi3l1 OE (*n = 5*) MIC mammary glands at 2 weeks post-induction. Ns: not significant, *p<0.05, **p<0.01, ***p<0.001 and ****p<0.0001 by One-Way Anova with Tukey's post-hoc test.

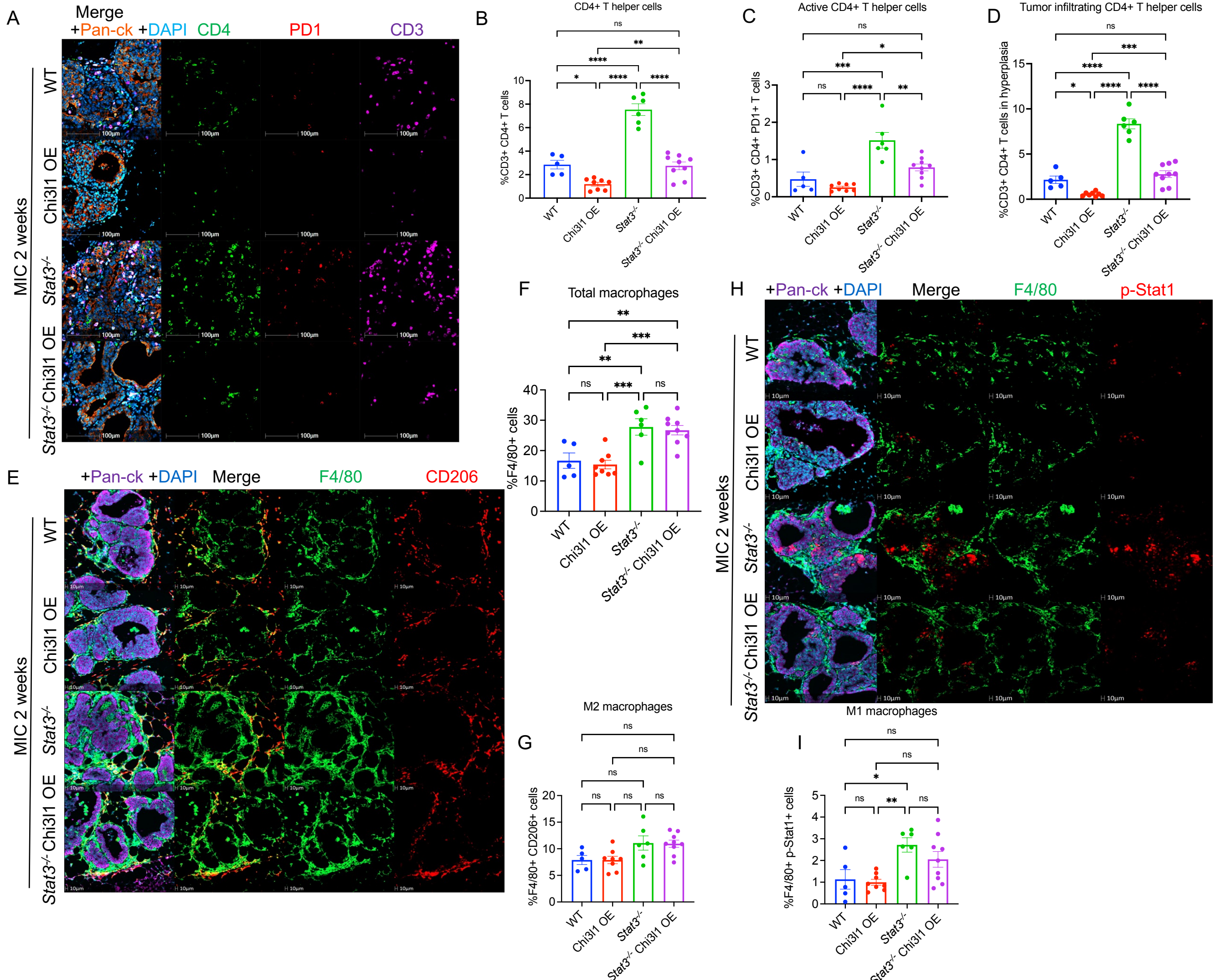

Supplementary Figure 4: Chi3l1 OE rewires the TIME.

(A) Staining of WT, Chi3l1 OE, *Stat3*$^{-/-}$ and *Stat3*$^{-/-}$ Chi3l1 OE MIC mammary glands at 2 weeks post-induction, for CD4, PD1, CD3, Pan-ck and DAPI. (B-D) Quantification of total CD3+CD4+, CD3+CD4+PD1+ and tumor-infiltrating CD3+CD4+ T cells in WT (*n* = 5), Chi3l1 OE (*n* = 8), *Stat3*$^{-/-}$ (*n* = 6) and *Stat3*$^{-/-}$ Chi3l1 OE (*n* = 9) MIC mammary glands. (E) Staining of WT, Chi3l1 OE, *Stat3*$^{-/-}$ and *Stat3*$^{-/-}$ Chi3l1 OE MIC mammary glands at 2 weeks post-induction, for F4/80, CD206, Pan-ck and DAPI. (F,G) Quantification of total F4/80+, and F4/80+CD206+ cells in WT (*n* = 5), Chi3l1 OE (*n* = 8), *Stat3*$^{-/-}$ (*n* = 6) and *Stat3*$^{-/-}$ Chi3l1 OE (*n* = 9) MIC mammary glands. (H) Staining of WT, Chi3l1 OE, *Stat3*$^{-/-}$ and *Stat3*$^{-/-}$ Chi3l1 OE MIC mammary glands at 2 weeks post-induction, for F4/80, p-Stat1, Pan-ck and DAPI. (I) Quantification of F4/80+p-Stat1+ cells in WT (*n* = 5), Chi3l1 OE (*n* = 8), *Stat3*$^{-/-}$ (*n* = 6) and *Stat3*$^{-/-}$ Chi3l1 OE (*n* = 9) MIC mammary glands. Ns: not significant, *p<0.05, **p<0.01, ***p<0.001 and ****p<0.0001 by One-Way Anova with Tukey's post-hoc test.

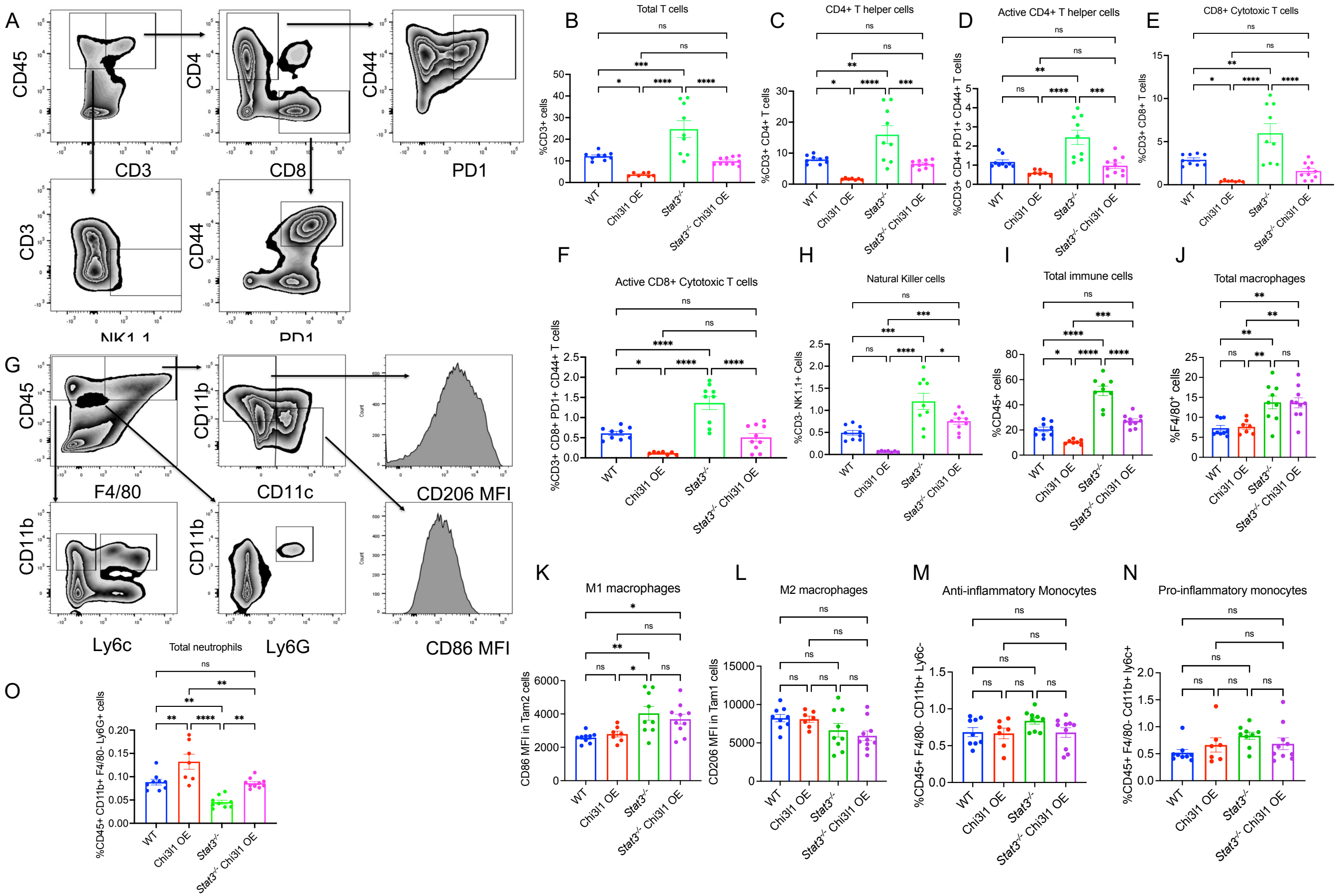

Supplementary Figure 5: FACS analysis confirms changes to the TIME.

(A) FACS gating strategy for lymphocytes in WT, Chi3l1 OE, *Stat3*$^{-/-}$ and *Stat3*$^{-/-}$ Chi3l1 OE MIC mammary glands at 2 weeks post-induction. Briefly, CD45+ cells were gated based on CD3 and divided based on CD8/CD4. The activation status of each T cell population was assessed through PD1 and CD44. NK cells were gated out of the CD45+CD3- population based on NK1.1. (B-F) Quantification of CD3+, CD3+CD4+, CD3+CD8+, CD3+CD8+PD1+CD44+, and CD3+CD4+PD1+CD44+ cells as percent of live cells in WT *(n* = 9*)*, Chi3l1 OE (*n* = 7), *Stat3*$^{-/-}$ (*n* = 9) and *Stat3*$^{-/-}$ Chi3l1 OE (*n* = 10) MIC mammary glands. (G) FACS gating strategy of monocytes, macrophages and neutrophils in WT, Chi3l1 OE, *Stat3*$^{-/-}$ and *Stat3*$^{-/-}$ Chi3l1 OE MIC mammary glands at 2 weeks post-induction. Briefly, CD45+ cells were sorted based on Ly6C and CD11b. Macrophages were gated out of the Ly6c- population based on F4/80 and divided into Tam1 (CD11b high, CD11c low) and Tam2 (CD11b low, CD11c high). CD206 expression levels (Mean Fluorescent Intensity, MFI) in Tam1 was used to measure M2 macrophages and CD86 MFI in Tam2 was used to measure M1 macrophages. Monocytes were gated out of CD45+F4/80- population based on expression of CD11b and Ly6c into anti-inflammatory (CD11b+Ly6c-) and pro-inflammatory (CD11b+Ly6c+). Neutrophils (Ly6G+) cells were gated out of CD11b+F4/80- cells. (H-O) Quantification of CD3-NK1.1+, CD45+, F4/80+, CD206 MFI in Tam1 cells (M2 macrophages), CD86 MFI in Tam2 cells (M1 macrophages), CD45+F4/80-CD11b+Ly6c- cells (Anti-inflammatory monocytes), CD45+F4/80-CD11b+Ly6c+ cells (Pro-inflammatory monocytes), and CD45+Cd11b+F4/80-Ly6G+ cells (neutrophils) as percent of live cells in WT *(n* = 9*)*, Chi3l1 OE *(n* = 7*)*, *Stat3*$^{-/-}$ (*n* = 9) and *Stat3*$^{-/-}$ Chi3l1 OE (*n* = 10) MIC mammary glands. Ns: not significant, *p<0.05, **p<0.01, ***p<0.001 and ****p<0.0001 by One-Way Anova with Tukey's post-hoc test.

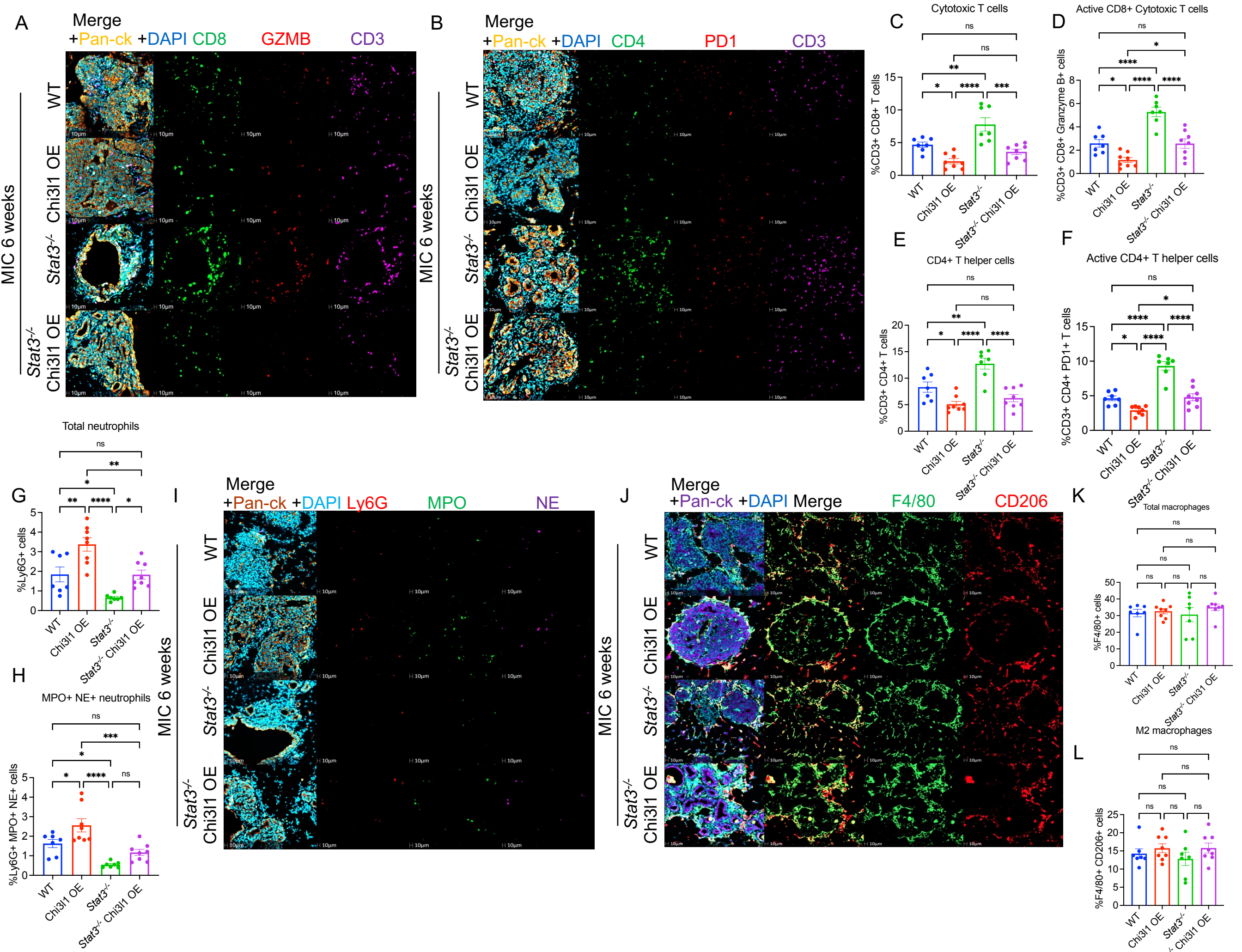

Supplementary Figure 6: Changes in the TIME were confirmed at 6-weeks post-induction
(A) Staining of WT, Chi3l1 OE, *Stat3*$^{-/-}$ and *Stat3*$^{-/-}$ Chi3l1 OE MIC mammary glands at 6 weeks post-induction using antibodies against CD8, Granzyme B, CD3, Pan-ck, and DAPI. (B) Staining of WT, Chi3l1 OE, *Stat3*$^{-/-}$ and *Stat3*$^{-/-}$ Chi3l1 OE MIC mammary glands at 6 weeks post induction using antibodies against CD4, PD1, CD3, Pan-ck and DAPI. (C-H) Quantification of CD3+CD8+ cells, CD3+CD8+GZMB+, CD3+CD4+ cells, CD3+CD4+PD1+ cells, Ly6G+ and Ly6G+MPO+NE+ cells in WT *(n* = 7*),* Chi3l1 OE (*n* = 8), *Stat3*$^{-/-}$ (*n* = 7) and *Stat3*$^{-/-}$ Chi3l1 OE *(n* = 8) MIC mammary glands at 6 weeks post-induction. (I) Staining of WT, Chi3l1 OE, *Stat3*$^{-/-}$ and *Stat3*$^{-/-}$ Chi3l1 OE MIC mammary glands at 6 weeks post-induction using antibodies against MPO, Ly6G, NE, Pan-ck, and DAPI. (J) Staining of WT, Chi3l1 OE, *Stat3*$^{-/-}$ and *Stat3*$^{-/-}$ Chi3l1 OE MIC mammary glands at 6 weeks post-induction using antibodies against F4/80, CD206, Pan-ck and DAPI. (K,L) Quantification of total F4/80+ and F4/80+CD206+ cells in WT (*n* = 7), Chi3l1 OE (*n* = 8), *Stat3*$^{-/-}$ (*n* = 7) and *Stat3*$^{-/-}$ Chi3l1 OE (*n* = 8) MIC mammary glands at 6 weeks post-induction. Ns: not significant, *p<0.05, **p<0.01, ***p<0.001 and ****p<0.0001 by One-Way Anova with Tukey's post-hoc test.

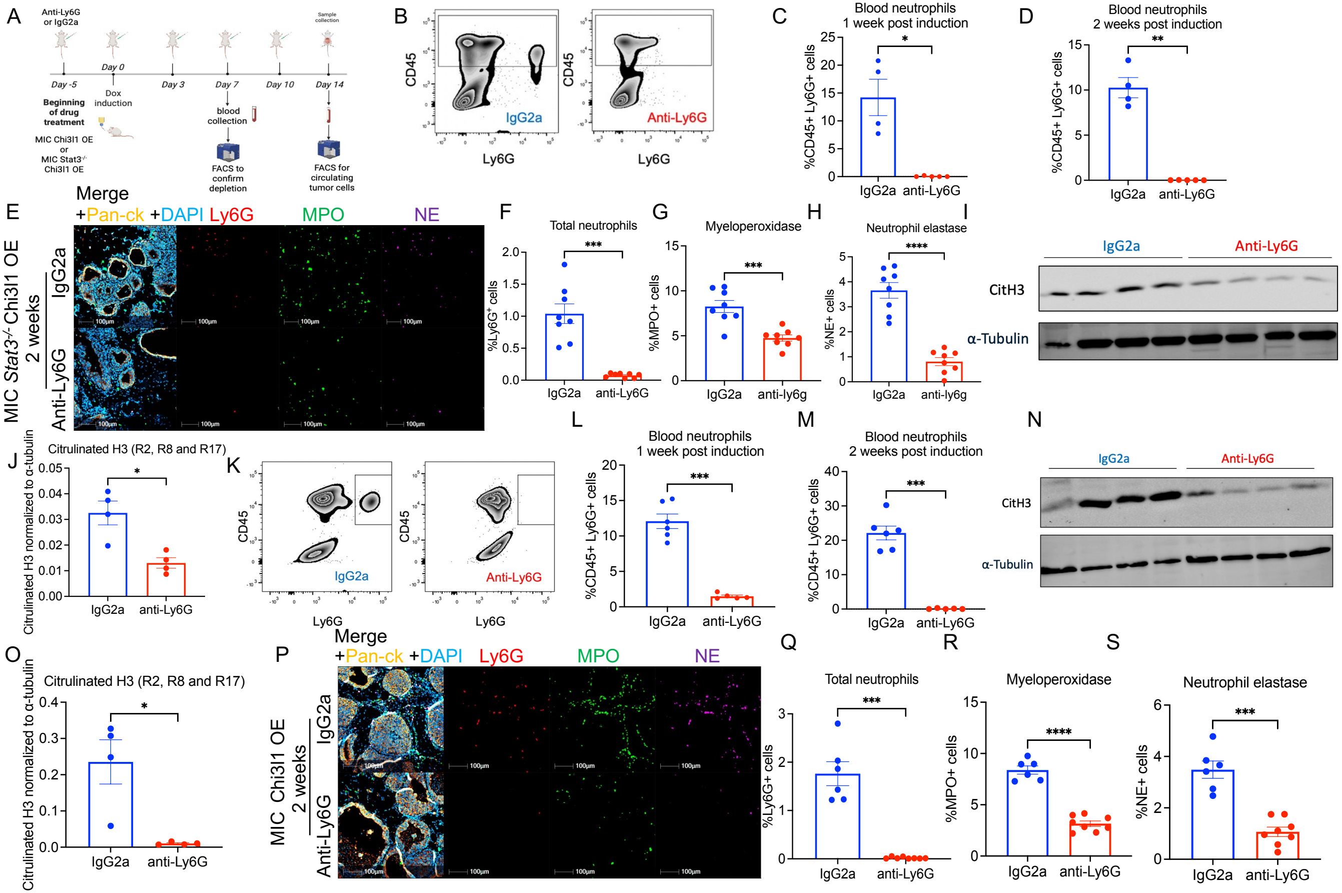

Supplementary figure 7: Confirmation of neutrophil depletion.
(A) Schematic for neutrophil depletion in MIC Stat3-deficient and proficient Chi3l1 OE mice using anti-Ly6G treatment, Created in BioRender. Muller, W. (2026) https://BioRender.com/viam8bd. (B) FACS for neutrophils in blood of *Stat3-/-* Chi3l1 OE MIC mice treated with anti-Ly6G or IgG2a at 7 days post-induction. CD45+ Leukocytes were sorted based on expression of Ly6G. The same strategy was used to detect blood neutrophils at 7 and 14-days post-induction. (C,D) Quantification of CD45+Ly6G+ cells as percent of live cells in the blood of *Stat3-/-* Chi3l1 OE MIC mice treated with anti-Ly6G antibody (*n* = 5) or IgG2a (*n* = 4) at 7 or 14-days post-induction. (E) Staining of mammary tissue from *Stat3-/-* Chi3l1 OE MIC mice treated with anti-Ly6G or IgG2a for 2 weeks, for Ly6G, MPO, NE, Pan-ck, and DAPI. (F-H) Quantification of Ly6G+, MPO+ and NE+ cells in IgG2a (*n* = 8) and anti-Ly6G (*n* = 8) treated *Stat3-/-* Chi3l1 OE MIC mammary glands. (I) Immunoblots for CitH3 and α-tubulin on IgG2a (*n* = 4) and anti-Ly6G (*n* = 4) treated *Stat3-/-* Chi3l1 OE MIC mammary glands at 2 weeks post-induction. (J) Quantification of CitH3 immunoblot normalized to α-tubulin. (K) FACS for neutrophils in blood of Chi3l1 OE MIC mice treated with anti-Ly6G or IgG2a at 7 days post-induction. (L,M) Quantification of CD45+Ly6G+ cells as percent of live cells in the blood of Chi3l1 OE MIC mice treated with anti-Ly6G antibody (*n* = 5) or IgG2a (*n* = 6) at 7 or 14-days post-induction. (N) Immunoblots for CitH3 and α-tubulin on IgG2a (*n* = 4) and anti-Ly6G (*n* = 4) treated Chi3l1 OE MIC mammary glands at 2 weeks post-induction. (O) Quantification of CitH3 immunoblot normalized to α-tubulin. (P) Staining of mammary tissue from Chi3l1 OE MIC mice treated with anti-Ly6G or IgG2a for 2 weeks, for Ly6G, MPO, NE, Pan-ck and DAPI. (Q-S) Quantification of Ly6G+, MPO+ and NE+ cells in IgG2a (*n* = 6) and anti-Ly6G (*n* = 8)-treated Chi3l1 OE MIC mammary glands. *p<0.05, **p<0.01, ***p<0.001 and ****p<0.0001 by unpaired Student's T test.

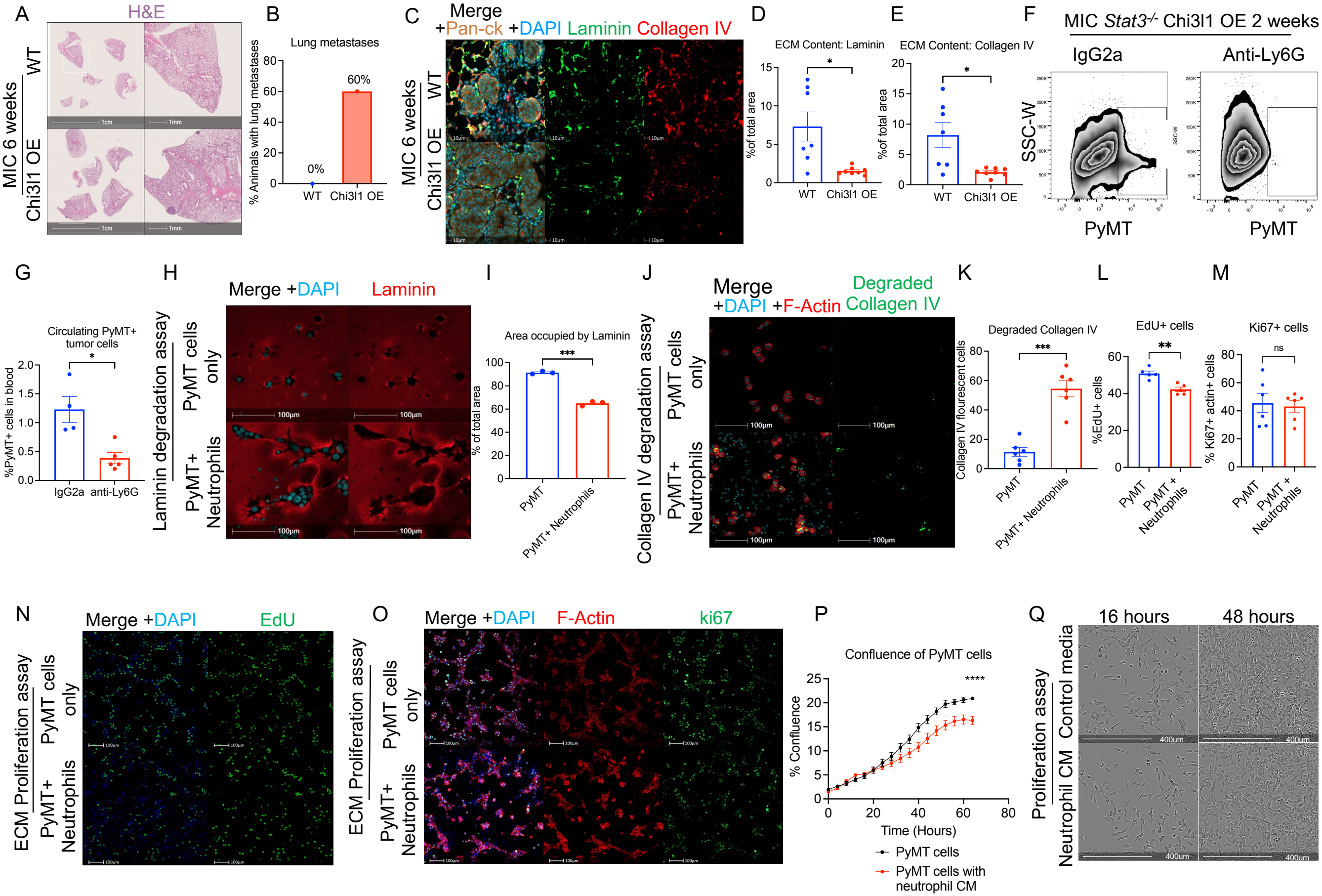

Supplementary figure 8: Neutrophils enhance Chi3l1-driven cancer cell invasion. (A) H&E staining of WT and Chi3l1 OE MIC lungs at 6 weeks post-induction. (B) Percentage of WT and Chi3l1 OE MIC mice with lung metastases at 6 weeks post-induction. (C) Staining of WT and Chi3l1 OE MIC mammary glands at 6 weeks post-induction, for laminin, collagen IV, Pan-ck and DAPI. (D,E) Quantification of area occupied by laminin or collagen IV in WT (n = 7) and Chi3l1 OE (n = 8) MIC mammary glands. (F) FACS for PyMT+ cells in the blood of *Stat3*$^{-/-}$ Chi3l1 OE MIC mice treated with IgG2a or anti-Ly6G antibody for 2 weeks. (G) Quantification of PyMT+ cells as percent of live cells in the blood of *Stat3*$^{-/-}$ Chi3l1 OE MIC mice treated with anti-Ly6G (n = 5) or IgG2a (n = 4). (H) Staining for laminin and DAPI on PyMT+ cells cultured alone or with neutrophils on synthetic ECM. (I) Quantification of area occupied by laminin in (H) (n = 3 technical replicates). (J) PyMT+ cells cultured alone or with neutrophils on fluorescently-labeled, degraded collagen IV ECM. (K) Quantification of degraded collagen IV in (J) (n = 6 technical replicates). (L,M) Quantification of EdU+ and F-actin+Ki67+ cells in (N,O) (n = 3 technical replicates). (N,O) Staining for EdU, Ki67, F-actin and DAPI on PyMT+ cells cultured alone or with neutrophils. (P) PyMT+ cell proliferation when cultured in neutrophil conditioned media (CM) or control media (n= *3* technical replicates). (Q) Representative images of PyMT+ cell proliferation assay when cultured in neutrophil CM or control media at 16 and 48 hours. Ns: not significant, *p<0.05, **p<0.01 and ***p<0.001 by unpaired Student's T test (for D,E,G,I,K, L,M and P) or One-Way Anova with Tukey's post-hoc test (for S).

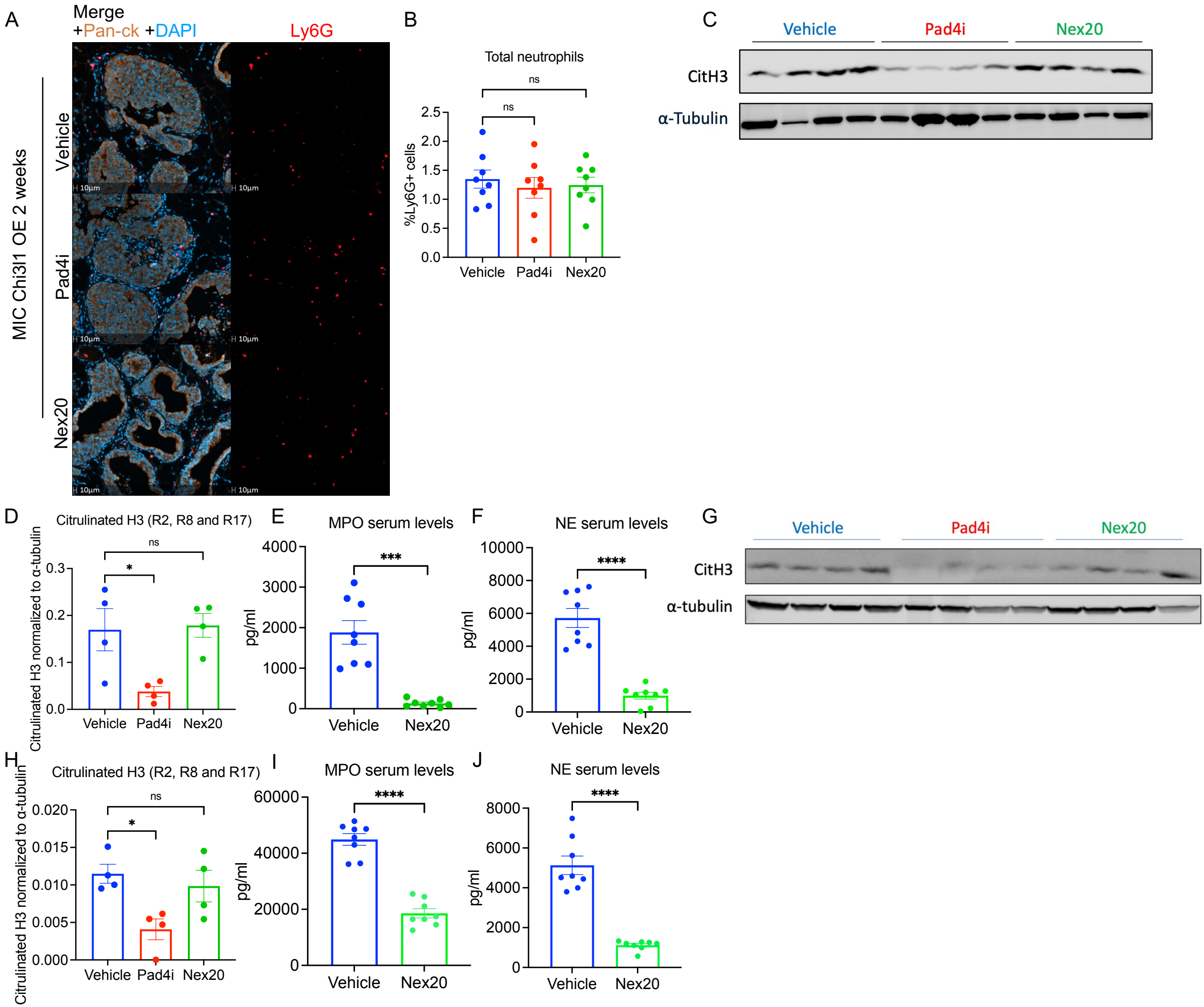

Supplementary figure 9: Validation of successful inhibition of NETosis and neutrophil degranulation.
(A) Staining of mammary tissue from MIC Chi3l1 OE mice treated with Pad4i, Nex20 or Vehicle control for 2 weeks, for Ly6G, Pan-ck and DAPI (blue). (B) Quantification of Ly6G+ cells in Pad4i (*n* = 8), Nex20 (*n* = 8) or Vehicle-treated (*n* = 8) MIC Chi3l1 OE mammary glands. (C) Immunoblots for CitH3 and α-tubulin on Vehicle (*n* = 4), Pad4i (*n* = 4) and Nex20 (*n* = 4)-treated Chi3l1 OE MIC mammary glands at 2 weeks post-induction. (D) Quantification of CitH3 immunoblot normalized to α-tubulin. (E,F) Elisa for serum MPO and NE from Vehicle (*n* = 8) and Nex20 (*n* = 8) treated Chi3l1 OE MIC mice at 2-weeks post induction. (G) Immunoblots for CitH3 and α-tubulin on Vehicle (*n* = 4), Pad4i (*n* = 4) and Nex20 (*n* = 4) treated Chi3l1 OE MIC mammary glands at 6-weeks post-induction. (H) Quantification of CitH3 immunoblot normalized to α-tubulin. (I,J) Elisa for serum MPO and NE from Vehicle (*n* = 8) and Nex20 (*n* = 8) treated Chi3l1 OE MIC mice at 6 weeks post-induction. Ns: Not significant, *p<0.05, ***p<0.001 and ****p<0.0001 calculated by One-Way Anova with Dunnett's post-hoc test (for B,D,H) or unpaired Student's T test (for E,F,I,J).

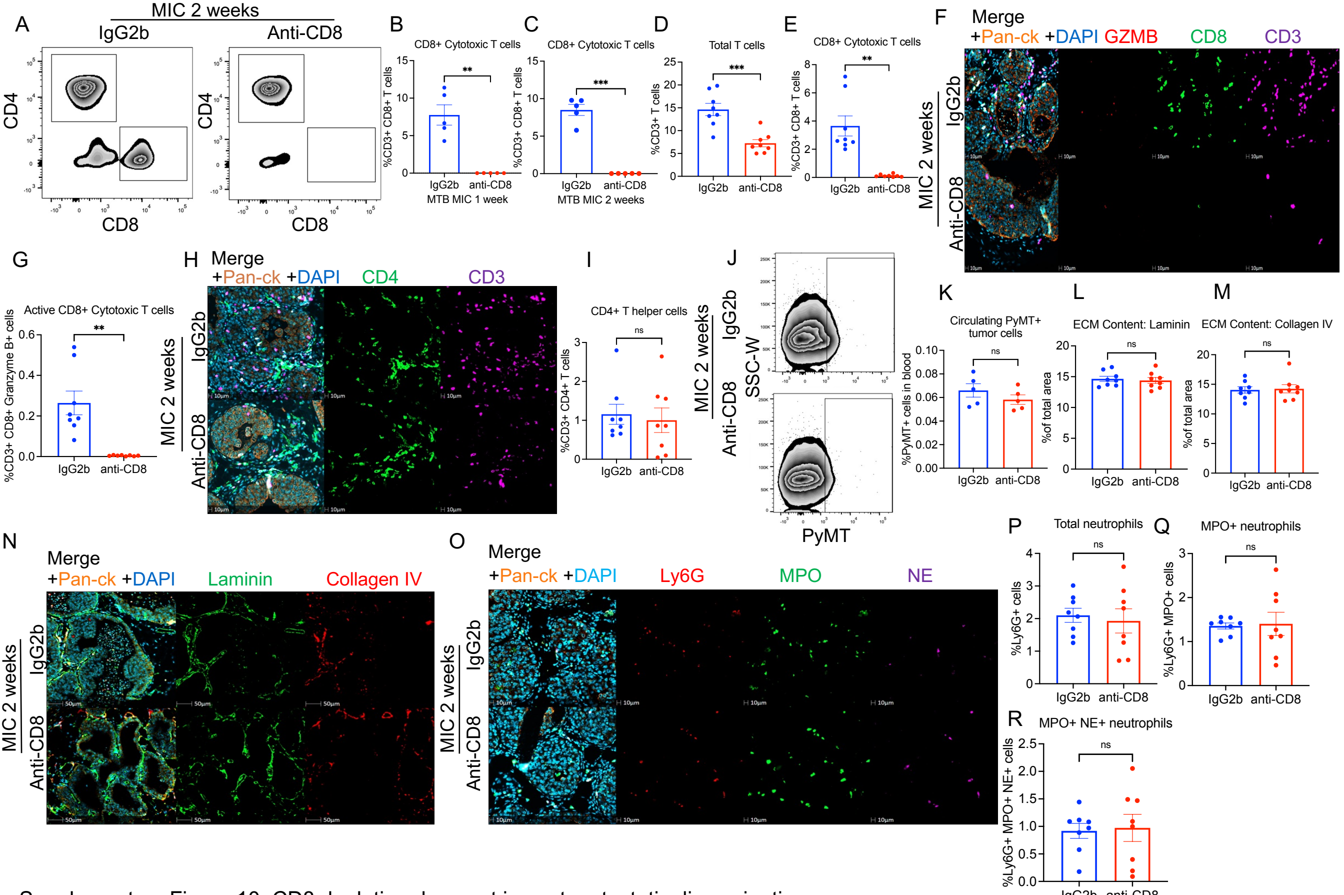

Supplementary Figure 10: CD8 depletion does not impact metastatic dissemination.
(A) FACS for T cells in blood of WT MIC mice treated with anti-CD8 or IgG2b at 7 days post induction. CD45+CD3+ cells were gated based on CD8/CD4. The same strategy was used to detect blood T cells at 7 and 14-days post-induction. (B,C) Quantification of CD45+CD3+CD8+ cells as percent of live cells in blood of WT MIC mice treated with anti-CD8 (*n* = 5) or IgG2b (*n* = 5) at 7 or 14-days post-induction. (D,E) Quantification of CD3+ and CD3+CD8+ in IgG2b (*n* = 8) and anti-CD8 (*n* = 8) treated WT MIC mammary glands at 2 weeks post-induction. (F) Staining of mammary tissue from WT MIC mice treated with anti-CD8 or IgG2b for 2 weeks, for GZMB, CD8, CD3, Pan-ck and DAPI. (G) Quantification of CD3+CD8+GZMB+ in IgG2b (*n* = 8) and anti-CD8 (*n* = 8) treated WT MIC mammary glands. (H) Staining of mammary tissue from WT MIC mice treated with anti-CD8 or IgG2b for 2 weeks, for CD4, CD3, Pan-ck and DAPI. (I) Quantification of CD3+CD4+ in IgG2b (*n* = 8) and anti-CD8 (*n* = 8) treated WT MIC mammary glands. (J) FACS for PyMT+ cells in the blood of WT MIC mice treated with anti-CD8 or IgG2b at 2 weeks post-induction. (K) Quantification of PyMT+ cells as percent of live cells in the blood of WT MIC mice treated with IgG2b *(n* = 5) or anti-CD8 *(n* = 5). (L,M) Quantification of area occupied by laminin or collagen IV in IgG2b (*n* = 5) and anti-CD8 (*n* = 5) treated WT MIC mammary glands at 2 weeks post-induction. Represented as percent of total mammary gland area. (N) Staining of mammary tissue from WT MIC mice treated with anti-CD8 or IgG2b for 2 weeks, for laminin, collagen IV, Pan-ck and DAPI. (O) Staining of mammary tissue from WT MIC mice treated with anti-CD8 or IgG2b for 2 weeks, for Ly6G, MPO, NE, Pan-ck and DAPI. (P-R) Quantification of Ly6G+, Ly6G+MPO+, and Ly6G+MPO+NE+ cells in IgG2b (*n* = 5) and anti-CD8 (*n* = 5) treated WT MIC mammary glands. Ns: Not significant, **p<0.01 and ***p<0.001 by unpaired Student's T test.

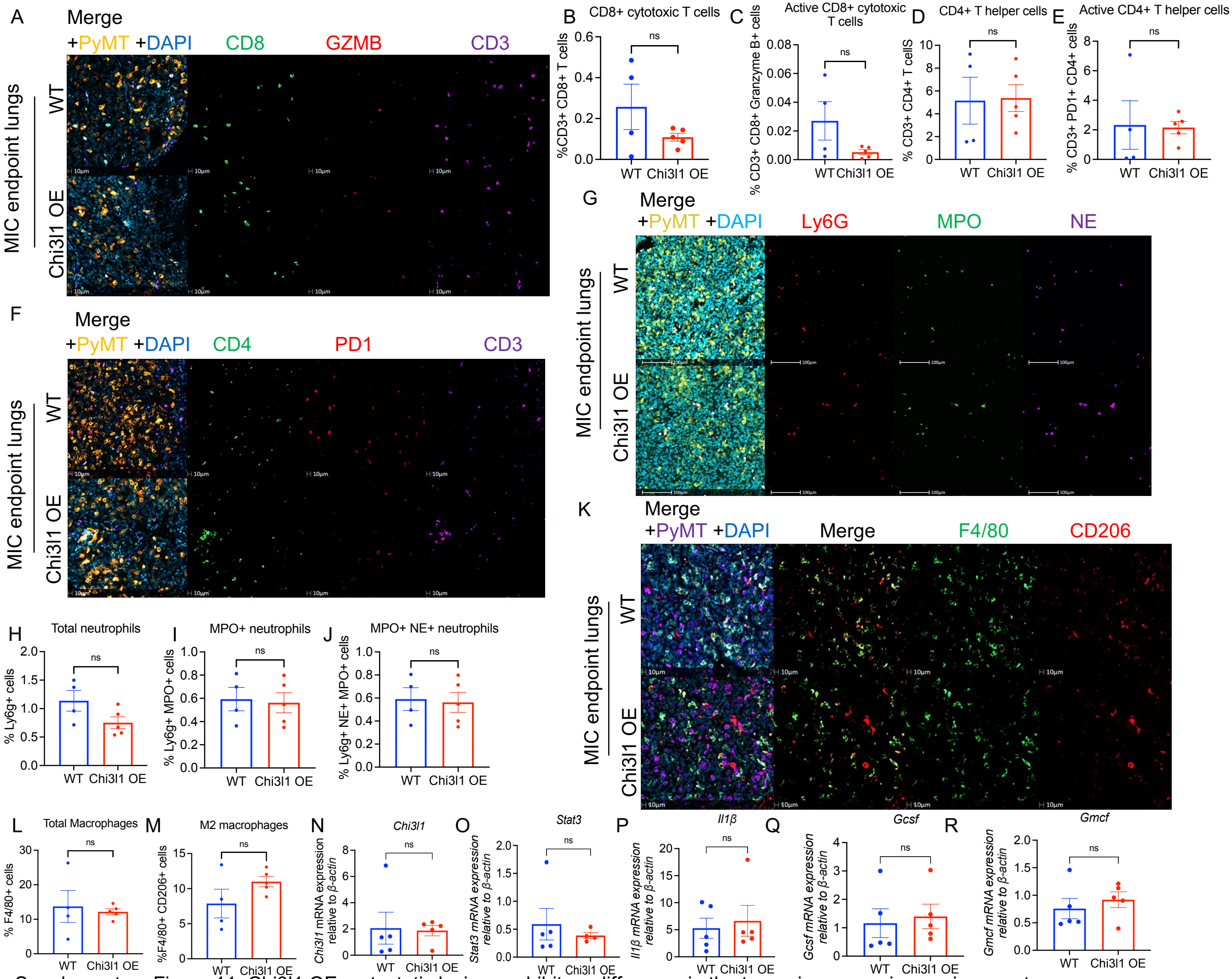

Supplementary Figure 11: Chi3l1 OE metastatic lesions exhibit no difference in the tumor immune microenvironment
(A) Staining of metastatic lung lesions from WT and Chi3l1 OE MIC mice for GZMB, CD8, CD3, PyMT, and DAPI. (B-E) Quantification of CD3+CD8, CD3+CD8+GZMB+, CD3+CD4+ and CD3+CD4+PD1+ in WT (*n* = 4) and Chi3l1 OE (*n* = 5) metastatic lung lesions. (F) Staining of metastatic lung lesions from WT and Chi3l1 OE MIC mice for CD4, PD1, CD3, PyMT and DAPI. (G) Staining of metastatic lung lesions from WT and Chi3l1 OE MIC mice for MPO, Ly6G, NE, PyMT, and DAPI. (H-J) Quantification of Ly6G+, Ly6G+MPO+, and Ly6G+MPO+NE+ cells in in WT (*n* = 4) and Chi3l1 OE (*n* = 5) metastatic lung lesions. (K) Staining of metastatic lung lesions from WT and Chi3l1 OE MIC mice for F4/80, CD206, PyMT and DAPI. (L,M) Quantification of F4/80+ and F4/80+CD206+ cells in in WT (*n* = 4) and Chi3l1 OE (*n* = 5) metastatic lung lesions. (N-R) qRT-PCR analysis of *Chi3l1, Stat3, Il1*β, *Gcsf* and *Gmcf* levels normalized to *β-actin* in WT (*n* = 5) and Chi3l1 OE metastatic lung lesions (*n* = 5). NS: Not significant, calculated by unpaired Student's T test.

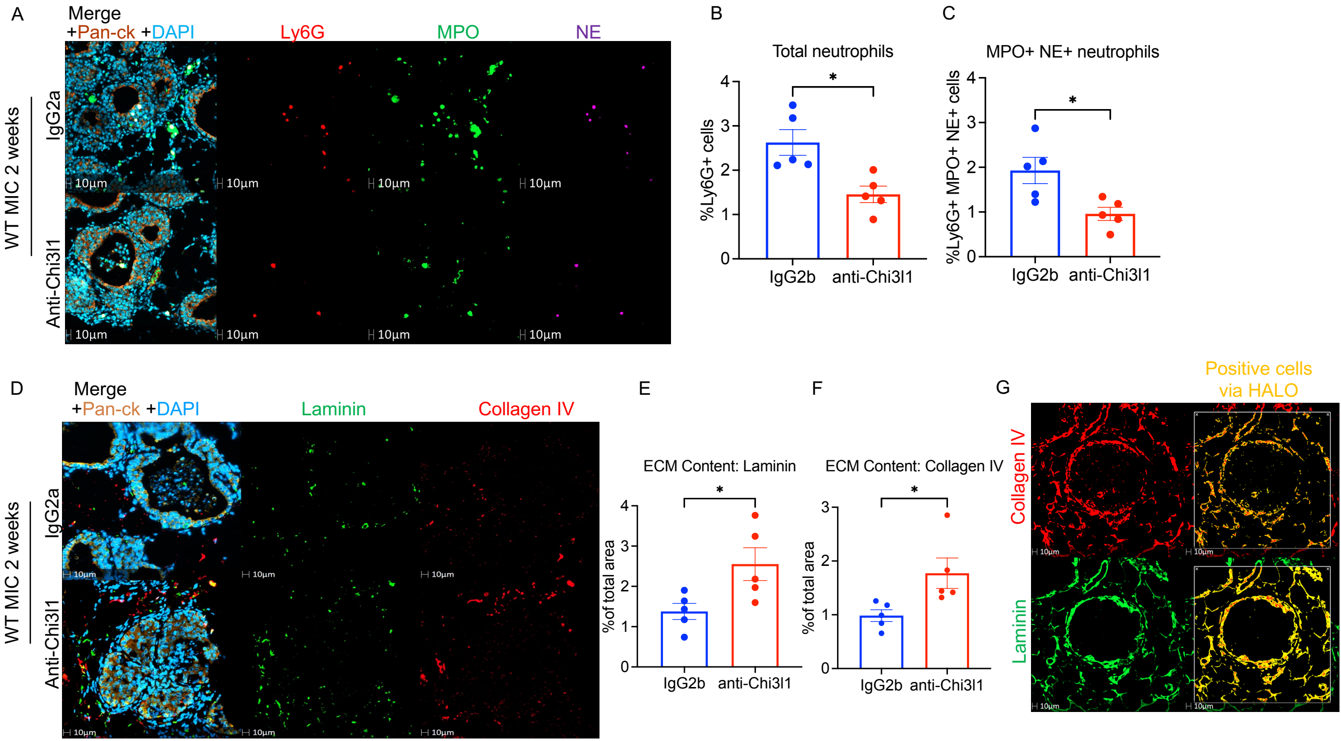

Supplementary figure 12: Neutralizing Chi3l1 restores ECM content.
(A) Staining of mammary tissue from WT MIC mice treated with anti-Chi3l1 neutralizing antibody or IgG2b for 2 weeks, for Ly6G, MPO, NE, Pan-ck and DAPI. (B,C) Quantification of Ly6G+, Ly6G+MPO+NE+ cells in IgG2b (*n* = 5) and anti-Chi3l1 (*n* = 5) treated WT MIC mammary glands. (D) Staining of mammary tissue from WT MIC mice treated with anti-Chi3l1 neutralizing antibody or IgG2b for 2 weeks, for laminin, collagen IV, Pan-ck and DAPI (blue). (E,F) Quantification of area occupied by laminin or collagen IV in IgG2b (*n* = 5) and anti-Chi3l1 (*n* = 5) treated WT MIC mammary glands, represented as percent of total mammary gland area (G) Representative example of the HALO teaching algorithm used to delineate the area occupied by laminin or collagen IV, determined by HALO. The same scoring system was applied to all analyzed samples in Figures 4, 6, 6 and supplemental figure 8, 10, and 12. *p<0.05, calculated by unpaired Student's T test.

A

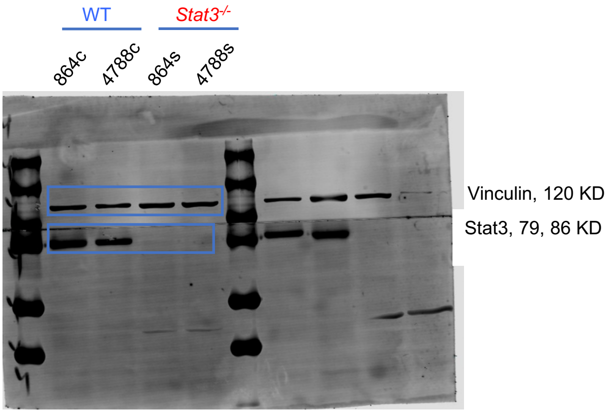

Supplementary figure 13: Full unedited gel for Supplementary Figure 1B
(A) Immunoblot for Stat3 and Vinculin on Stat3 WT (864c, 4788c) and *Stat3*$^{-/-}$ (864s, 4788s) cell lines.

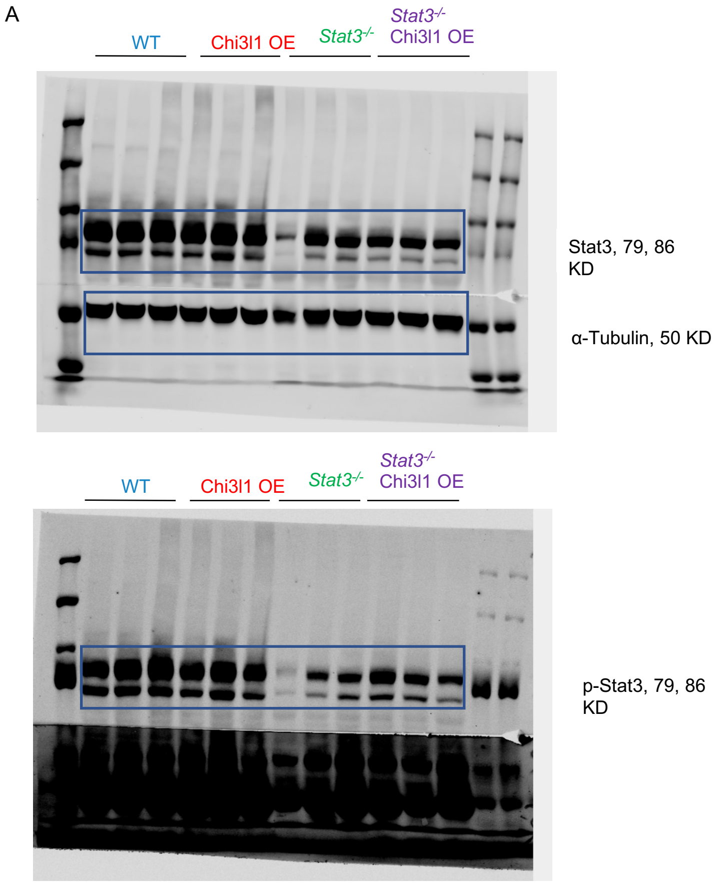

Supplementary figure 14: Full unedited gel for Supplementary Figure 2A
(A) Immunoblots for Stat3, p-Stat3 and α-tubulin on WT (n = 3), Chi3l1 OE (n = 3), *Stat3*$^{-/-}$ (n = 3) and *Stat3*$^{-/-}$ Chi3l1 OE (n = 3) MIC mammary glands at 2 weeks post induction. The same immunoblot is presented twice for separate channels.

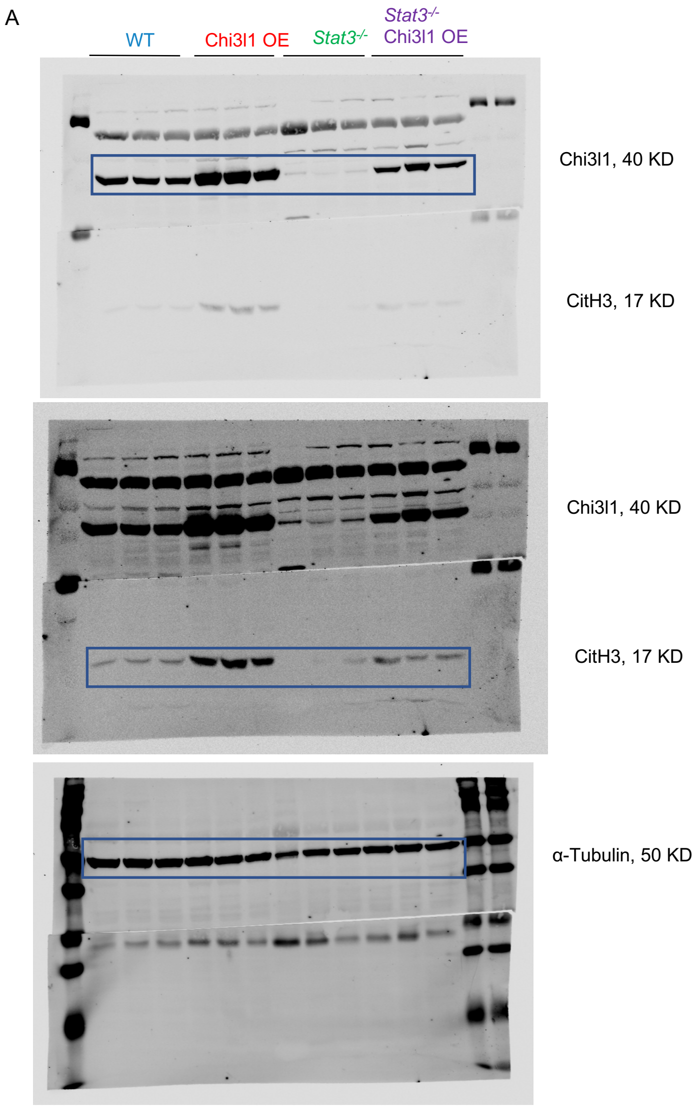

Supplementary figure 15: Full unedited gel for Supplementary Figure 2A and Figure 2L
(A) Immunoblots for Chi3l1, CitH3 and α-tubulin on WT (n = 3), Chi3l1 OE (n = 3), *Stat3*$^{-/-}$ (n = 3) and *Stat3*$^{-/-}$ Chi3l1 OE (n = 3) MIC mammary glands at 2 weeks post induction. The same immunoblot is presented three times for separate channels at different intensities. The same blot was used to assess levels of Chi3l1 and CitH3. This blot was ran at the same time as the blots presented in Supplementary Figure 6 looking at Stat3 and p-Stat3.

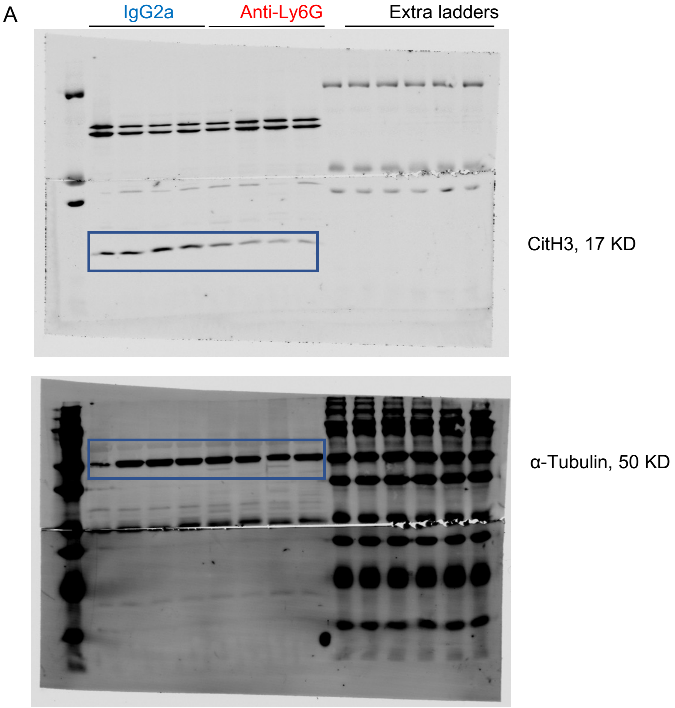

Supplementary figure 16: Full unedited gel for Supplementary Figure 7I
(A) Immunoblots for CitH3 and α-tubulin on IgG2a (n = 4) and anti-Ly6G (n = 4) treated *Stat3*$^{-/-}$ Chi3l1 OE MIC mammary glands at 2 weeks post induction. The same immunoblot is presented twice for separate channels.

A

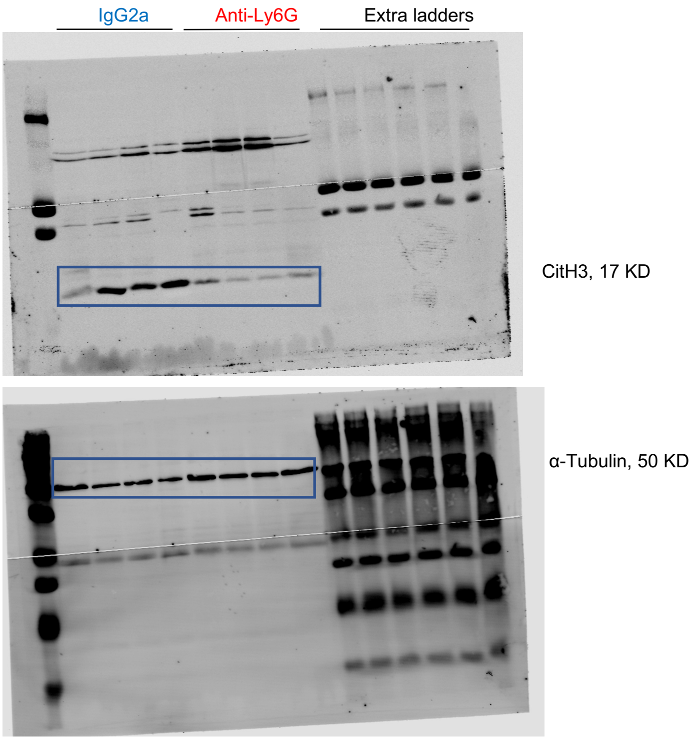

Supplementary figure 17: Full unedited gel for Supplementary Figure 7N
(A) Immunoblots for CitH3 and α-tubulin on IgG2a (n = 4) and anti-Ly6G (n = 4) treated Chi3l1 OE MIC mammary glands at 2 weeks post induction. The same immunoblot is presented twice for separate channels.

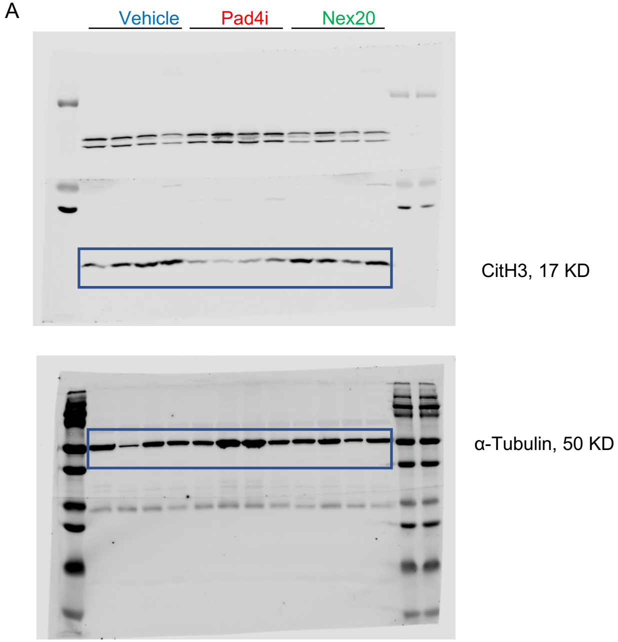

Supplementary figure 18: Full unedited gel for Supplementary Figure 9C
(A) Immunoblots for CitH3 and α-tubulin on Vehicle (*n* = 4), Pad4i (*n* = 4) and Nex20 (*n* = 4) treated Chi3l1 OE MIC mammary glands at 2 weeks post induction. The same immunoblot is presented twice for separate channels.

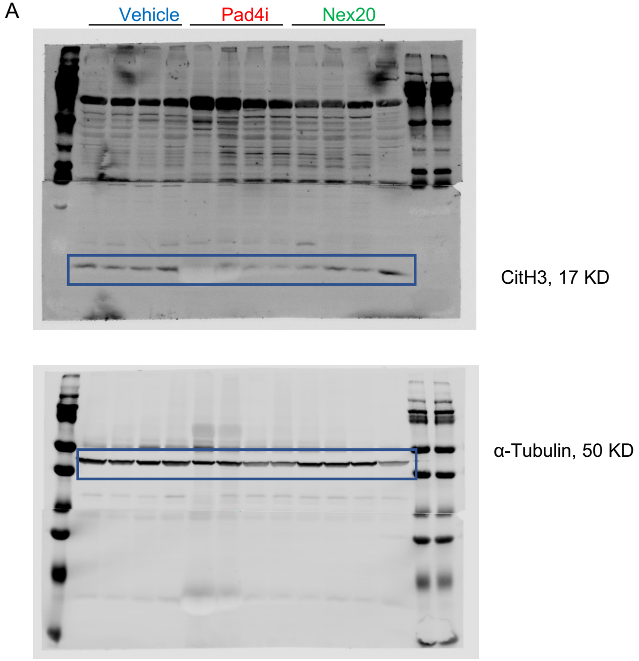

Supplementary figure 19: Full unedited gel for Supplementary Figure 9G
(A) Immunoblots for CitH3 and α-tubulin on Vehicle (*n* = 4), Pad4i (*n* = 4) and Nex20 (*n* = 4) treated Chi3l1 OE MIC mammary glands at 6 weeks post induction. The same immunoblot is presented twice for separate channels.

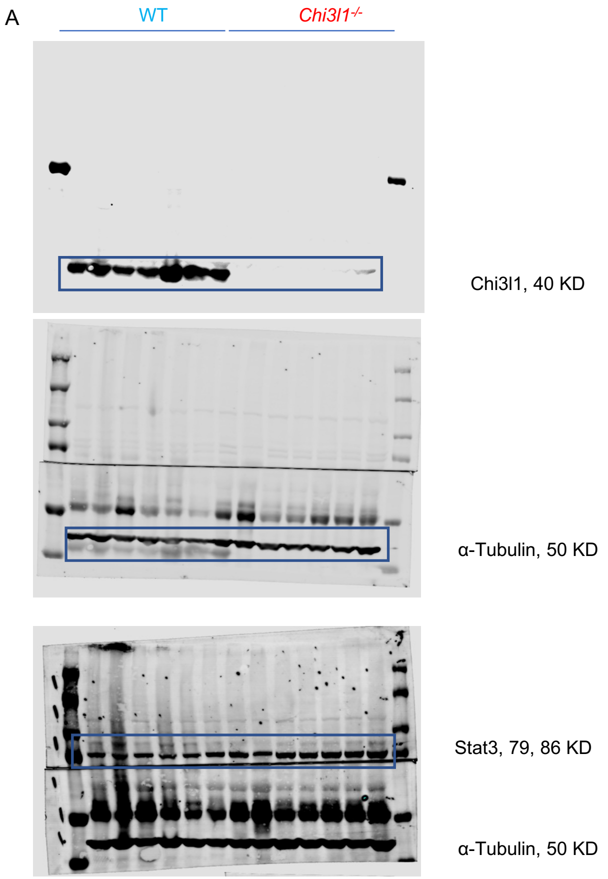

Supplementary figure 20: Full unedited gel for Supplementary Figure 6A
(A) Immunoblots for Chi3l1, Stat3, and α-tubulin on WT (n = 7), *Chi3l1*$^{-/-}$ (n = 6) MIC mammary glands at mammary tumor endpoint. The same immunoblot is presented twice for separate channels.
