# Supplementary material for: The CHI3L1-neutrophil axis drives immune suppression and breast cancer metastatic dissemination
Source: JCI Insight. 2026 Feb 3;11(6):e199307. doi: 10.1172/jci.insight.199307 (PMC13043092; doi:10.1172/jci.insight.199307)
Supplement: Unedited blot and gel images [file jciinsight-11-199307-s293.pdf]

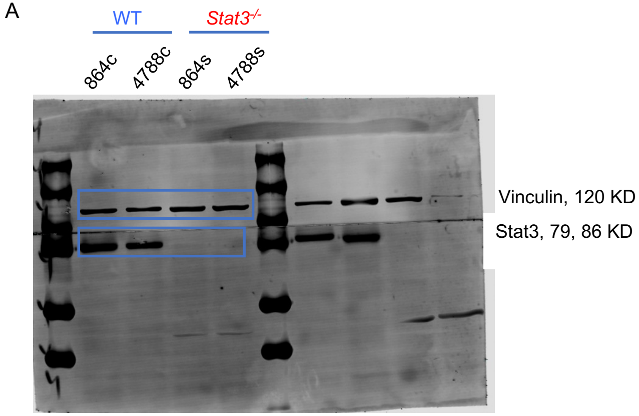

Supplementary figure 13: Full unedited gel for Supplementary Figure 1B
(A) Immunoblot for Stat3 and Vinculin on Stat3 WT (864c, 4788c) and *Stat3*$^{-/-}$ (864s, 4788s) cell lines.

A

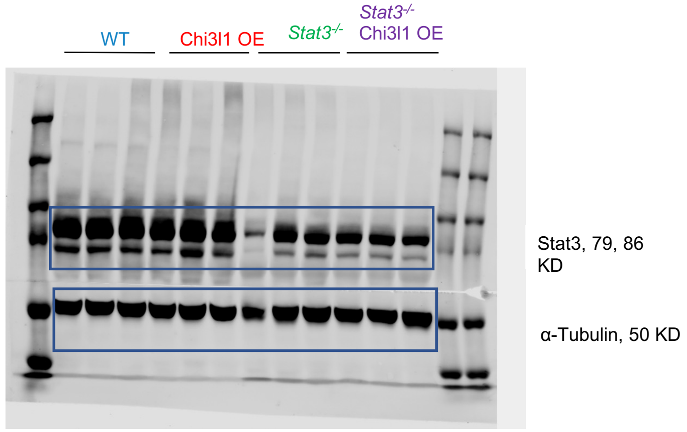

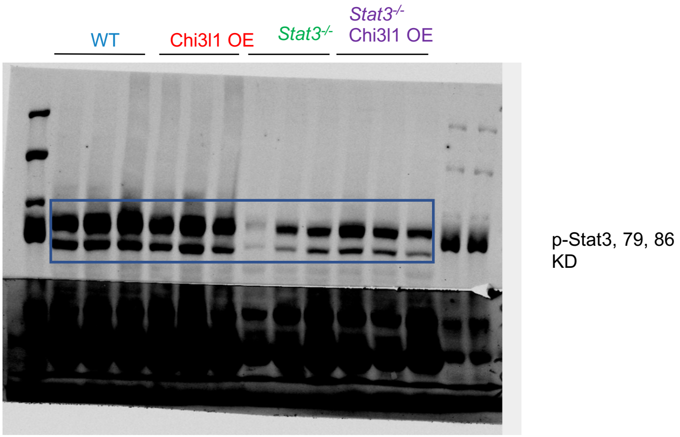

Supplementary figure 14: Full unedited gel for Supplementary Figure 2A
(A) Immunoblots for Stat3, p-Stat3 and α-tubulin on WT (n = 3), Chi3l1 OE (n = 3), *Stat3*$^{-/-}$ (n = 3) and *Stat3*$^{-/-}$ Chi3l1 OE (n = 3) MIC mammary glands at 2 weeks post induction. The same immunoblot is presented twice for separate channels.

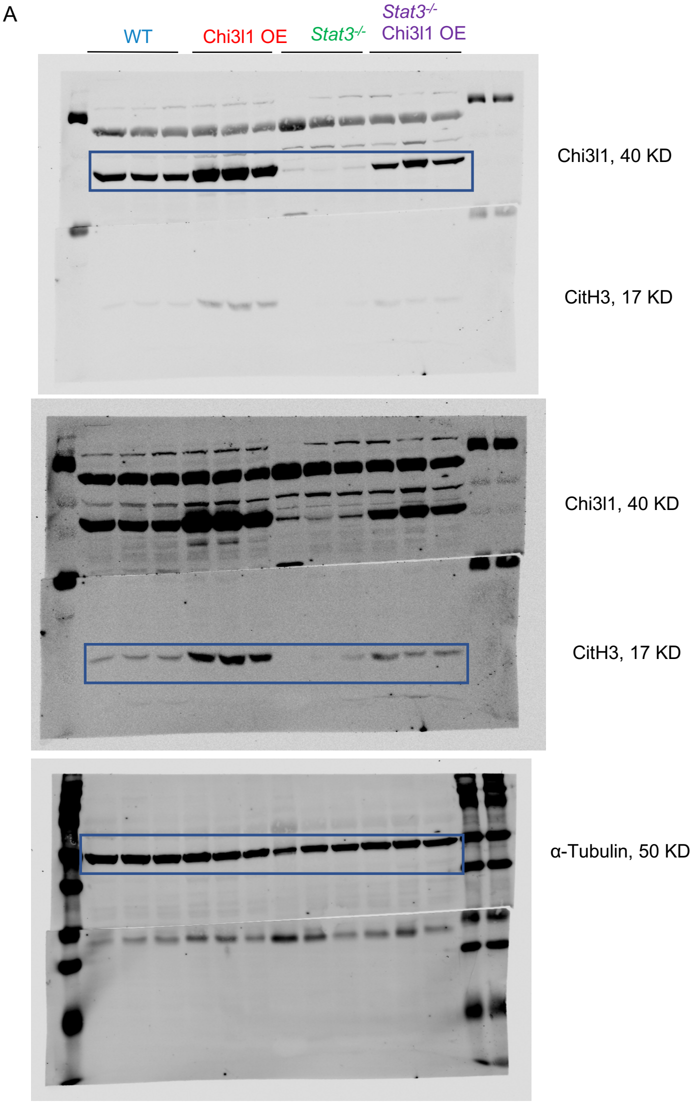

Supplementary figure 15: Full unedited gel for Supplementary Figure 2A and Figure 2L
(A) Immunoblots for Chi3l1, CitH3 and α-tubulin on WT (*n* = 3), Chi3l1 OE (*n* = 3), *Stat3*$^{-/-}$ (*n* = 3) and *Stat3*$^{-/-}$ Chi3l1 OE (*n* = 3) MIC mammary glands at 2 weeks post induction. The same immunoblot is presented three times for separate channels at different intensities. The same blot was used to assess levels of Chi3l1 and CitH3. This blot was ran at the same time as the blots presented in Supplementary Figure 6 looking at Stat3 and p-Stat3.

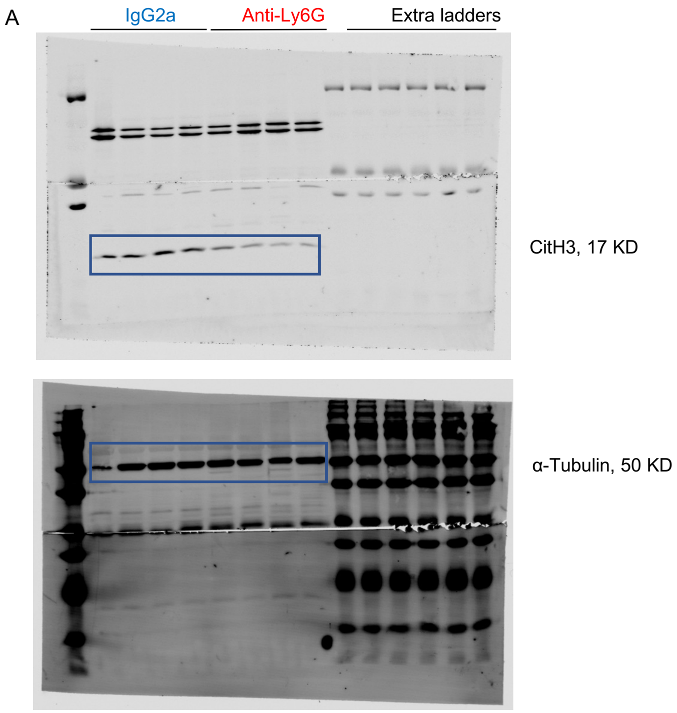

Supplementary figure 16: Full unedited gel for Supplementary Figure 7I
(A) Immunoblots for CitH3 and α-tubulin on IgG2a (*n* = 4) and anti-Ly6G (*n* = 4) treated *Stat3*$^{-/-}$ Chi3l1 OE MIC mammary glands at 2 weeks post induction. The same immunoblot is presented twice for separate channels.

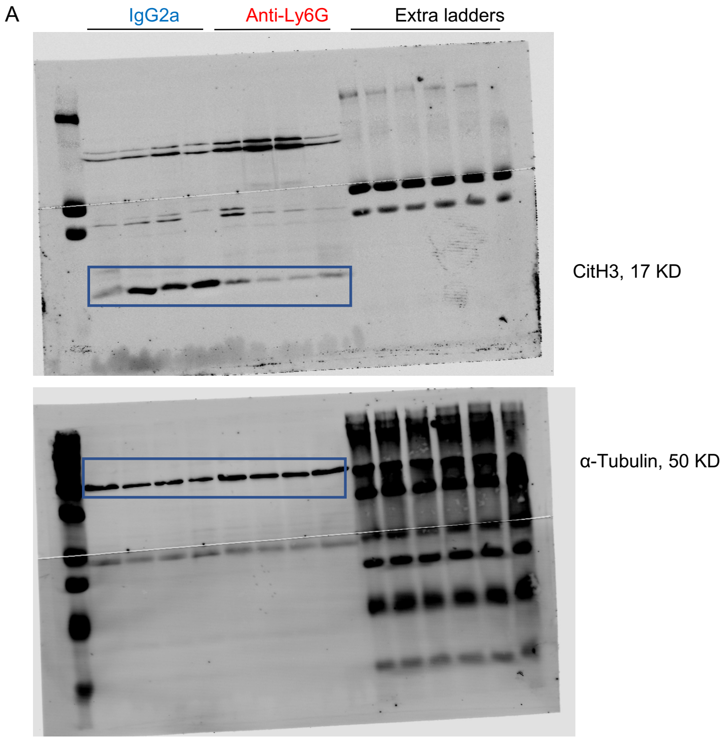

Supplementary figure 17: Full unedited gel for Supplementary Figure 7N
(A) Immunoblots for CitH3 and α-tubulin on IgG2a (*n* = 4) and anti-Ly6G (*n* = 4) treated Chi3l1 OE MIC mammary glands at 2 weeks post induction. The same immunoblot is presented twice for separate channels.

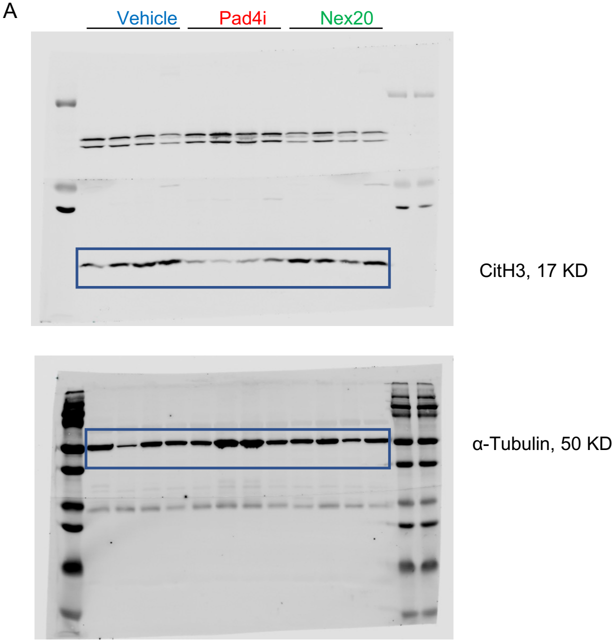

Supplementary figure 18: Full unedited gel for Supplementary Figure 9C
(A) Immunoblots for CitH3 and α-tubulin on Vehicle (*n* = 4), Pad4i (*n* = 4) and Nex20 (*n* = 4) treated Chi3l1 OE MIC mammary glands at 2 weeks post induction. The same immunoblot is presented twice for separate channels.

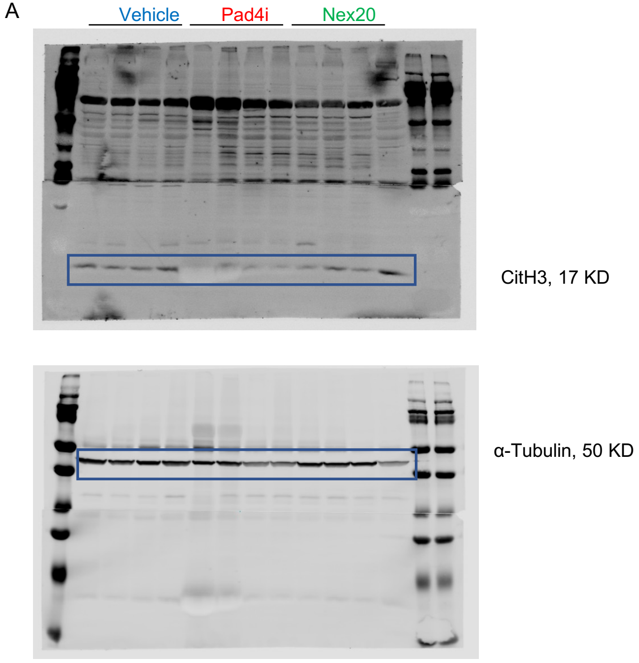

Supplementary figure 19: Full unedited gel for Supplementary Figure 9G
(A) Immunoblots for CitH3 and α-tubulin on Vehicle (*n* = 4), Pad4i (*n* = 4) and Nex20 (*n* = 4) treated Chi3l1 OE MIC mammary glands at 6 weeks post induction. The same immunoblot is presented twice for separate channels.

A

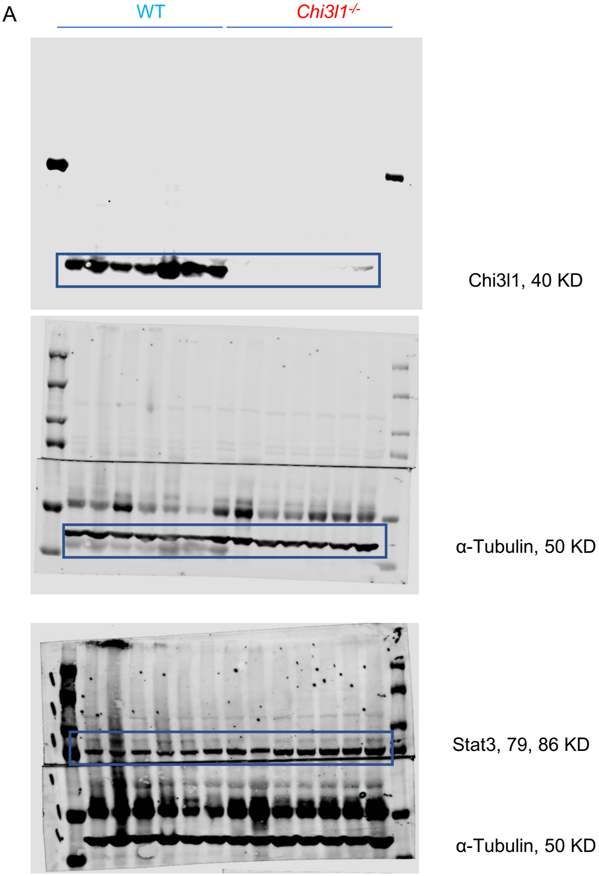

Supplementary figure 20: Full unedited gel for Supplementary Figure 6A
(A) Immunoblots for Chi3l1, Stat3, and α-tubulin on WT (n = 7), *Chi3l1*$^{-/-}$ (n = 6) MIC mammary glands at mammary tumor endpoint. The same immunoblot is presented twice for separate channels.
